# The cognitive model of negative symptoms: a systematic review and meta-analysis of the dysfunctional belief systems associated with negative symptoms in schizophrenia spectrum disorders

**DOI:** 10.1017/S0033291724003325

**Published:** 2025-02-05

**Authors:** Sarah Saperia, Joanne Plahouras, Michael Best, Sean Kidd, Konstantine Zakzanis, George Foussias

**Affiliations:** 1 Schizophrenia Division and Campbell Family Mental Health Research Institute, Centre for Addiction and Mental Health, Toronto, ON, Canada; 2 Department of Psychology, University of Toronto Scarborough, Toronto, ON, Canada; 3 Slaight Family Centre for Youth in Transition, Centre for Addiction and Mental Health, Toronto, ON, Canada; 4 Department of Psychiatry, Temerty Faculty of Medicine, University of Toronto, Toronto, ON, Canada; 5 Institute of Medical Science, University of Toronto, Toronto, Canada

**Keywords:** schizophrenia, negative symptoms, cognitive model, dysfunctional beliefs, meta-analysis, diminished motivation

## Abstract

**Background:**

The hypothesized cognitive model of negative symptoms, proposed nearly twenty years ago, is the most prevalent psychological framework for conceptualizing negative symptoms in schizophrenia spectrum disorders (SSDs). The aim of this study was to comprehensively validate the model for the first time, specifically by quantifying the relationships between negative symptom severity and all related dysfunctional beliefs.

**Methods:**

A systematic search was conducted using MEDLINE and PsychINFO, supplemented by manual reviews of reference lists and Google Scholar. Eligible studies were peer-reviewed with data on the direct cross-sectional association between negative symptoms and at least one relevant dysfunctional belief in SSD patients. Screening and data extraction were completed by independent reviewers. Random-effects meta-analyses were performed to pool effect size estimates of *z*-transformed Pearson’s *r* correlations. Moderators of these relationships, as well as subset analyses for negative symptom domains and measurement instruments, were also assessed.

**Results:**

Significant effects emerged for the relationships between negative symptoms and defeatist performance beliefs (k = 38, n = 2808), r = 0.23 (95% CI, 0.18–0.27), asocial beliefs (k = 8, n = 578), r = 0.21 (95% CI, 0.12–0.28), low expectancies for success (k = 55, n = 5664), r = −0.21 (95% CI, −0.15 – −0.26), low expectancies for pleasure (k = 5, n = 249), r = −0.19 (95% CI, −0.06 – −0.31), and internalized stigma (k = 81, n = 9766), r = 0.17 (95% CI, 0.12–0.22), but not perception of limited resources (k = 10, n = 463), r = 0.08 (95% CI, −0.13 – 0.27).

**Conclusions:**

This meta-analysis provides support for the cognitive model of negative symptoms. The identification of specific dysfunctional beliefs associated with negative symptoms is essential for the development of precision-based cognitive-behavioral interventions.

## Introduction

Negative symptoms, characterized by impaired motivation and emotional expression, are core features of schizophrenia spectrum disorders (SSDs) and are among the most reliable predictors of poor functional outcomes in affected individuals (Foussias & Remington, 2010). Nearly 20 years ago, Rector, Beck, and colleagues published seminal works on the cognitive model of negative symptoms, theorizing from a biopsychosocial perspective that negative symptoms represent maladaptive responses to dysfunctional beliefs, thought to emerge following life difficulties and setbacks that occur throughout the evolution of the disorder (Beck & Rector, 2005; Rector, Beck, & Stolar, 2005). Briefly, the model posits a set of unhelpful or dysfunctional belief systems that are underscored by common themes of ‘What’s the point?,’ ‘It’s not worth it,’ and ‘I can’t handle it.,’ including defeatist performance beliefs, asocial beliefs, low expectancies for success, low expectancies for pleasure, internalized stigma, and perception of limited resources. More specifically, defeatist performance beliefs refer to overgeneralized or rigid negative beliefs about performing goal-directed activity (e.g. ‘If you cannot do something well, there is little point in doing it at all’ (Weissman & Beck, 1978); asocial beliefs are aversive and/or apathetic attitudes towards social engagement and affiliation (e.g. ‘Having close friends is not as important as most people say’ (Grant & Beck, 2010)); low expectancies for success and pleasure, are characterized by generally negative expectations and low confidence for experiencing success or pleasure, respectively; internalized stigma in this context refers to self-limiting beliefs due to identification with negative stigmatizing views of mental illness (e.g. ‘What do you expect, I’m mentally ill’ (Rector et al., 2005); and perception of limited resources reflects the subjective sense of having diminished or insufficient physical and/or mental internal resources to expend effort or complete tasks. Importantly, these beliefs are putatively related to the development, expression, and maintenance of negative symptoms, and ostensibly represent mechanistic targets for psychological interventions such as cognitive behavioral therapy (CBT), which has thus far demonstrated fairly mixed results for its efficacy in treating negative symptoms (Cella et al., 2023).

As research on the cognitive model of negative symptoms has proliferated and evolved over the past two decades, it is imperative to synthesize this clinically rich body of work, and for the first time since its inception, comprehensively validate the hypothesized model. This approach may also serve to highlight a broad range of potential psychotherapeutic targets that can be translated into optimized CBT interventions for negative symptoms. Thus, a systematic review and meta-analysis were undertaken to empirically quantify the relationships between negative symptoms and each of the six primary dysfunctional beliefs postulated within the model: defeatist performance beliefs, asocial beliefs, low expectancies for success, low expectancies for pleasure, internalized stigma, and perception of limited resources.

## Methods

This review was registered with PROSPERO (CRD42021245467), completed in Covidence, and followed PRISMA guidelines (Moher, Liberati, Tetzlaff, Altman, & Group, 2009).

### Search strategy

An electronic search was conducted on MEDLINE and PsycINFO, from inception through January 2024. A broad search strategy was developed in consultation with an academic librarian (see supplementary methods). We also conducted a manual review of reference lists and Google Scholar for additional articles.

### Selection criteria

Titles and abstracts were independently screened for relevance (S.S., J.P., G.F.), after which full-text articles were assessed for eligibility using the following inclusion criteria: 1) written in English; 2) majority of sample (≥85%) diagnosed with non-affective SSDs, excluding schizotypal personality disorder or clinically high risk/prodrome (if <85%, study authors were contacted to request data with ineligible patients excluded); 3) includes valid measure of negative symptoms (global or subdomains); 4) includes measure of at least one relevant dysfunctional belief; 5) original data published in a peer-reviewed journal. For studies containing overlapping samples, the study with data for the larger sample size was included. Studies were also required to report statistics on the direct relationship between negative symptoms and dysfunctional beliefs, with sufficient data to estimate an effect size. Where otherwise eligible studies did not report this data, the author was contacted to request it. Only baseline cross-sectional data was included.

To enable a comprehensive review, we considered all instruments that appeared to evaluate relevant beliefs. Measures were only included if they assessed cognitions (i.e. beliefs, attitudes, expectancies); in the case of asocial beliefs and low expectancies for pleasure, we excluded instruments tapping into hedonic experiences (i.e. social anhedonia and anticipatory pleasure, respectively). Measures of stigma experiences or perceived societal stigma were also excluded. The final decision for inclusion was made by S.S. and G.F. based on consideration of the instrument’s use in other studies, as well as face validity in relation to the theoretical model.

### Data extraction

In addition to effect size statistics, study characteristics including sample size, country of origin, mean age, % male, diagnoses, illness duration, and negative symptom severity were extracted. Study authors were contacted to request this data if unpublished.

### Quality assessment

The methodological quality of studies was assessed using an adapted version of the Quality Assessment Tool for Quantitative Studies (Thomas, Ciliska, Dobbins, & Micucci, 2004), with sections pertaining to randomized controlled trials removed and only relevant indicators of quality included (see supplementary methods).

### Data analysis

Six meta-analyses were conducted to quantify the relationship between overall negative symptoms and each of the following dysfunctional beliefs: defeatist performance beliefs, asocial beliefs, low expectancies for success, low expectancies for pleasure, internalized stigma, and perception of limited resources. The direction of the reported correlation coefficient was reverse-coded, where necessary, so that an association between more severe negative symptoms and greater endorsement of dysfunctional beliefs is indicated by: 1) a positive correlation for defeatist performance beliefs, asocial beliefs, internalized stigma, and perception of limited resources; and 2) a negative correlation for expectancies for success and expectancies for pleasure to reflect more negative (i.e. lower) expectations.

All analyses were performed using the ‘metafor’ package in R software for statistical computation (R Core Team, 2013; Viechtbauer, 2010). Pearson’s *r* correlation coefficient was used for effect sizes. Two studies (Beck, Grant, Huh, Perivoliotis, & Chang, 2013; Bentall et al., 2010) used t-tests to compare dysfunctional beliefs in participants with high versus low negative symptoms, which were converted from Cohen’s *d* to *r* (Rosenthal, 1991). Spearman correlations were included in analyses when Pearson’s correlations could not be obtained.

Random-effects models with restricted maximum-likelihood estimators were used to pool effect size estimates of *z*-transformed Pearson’s *r* correlations. The primary analyses assessed the relationships between dysfunctional beliefs and global negative symptoms. If only subscale correlations were reported for either negative symptoms or dysfunctional beliefs instead of their total scores, they were averaged using Fisher’s *r*-to-*z* transformation to estimate an overall effect size. Missing data for nonsignificant correlations were imputed with a zero. Subset analyses were also conducted for the negative symptom subdomains of diminished motivation (i.e. avolition/apathy, anhedonia, and asociality) and diminished emotional expression (i.e. blunted affect and alogia), as well as the different measurement instruments used for both negative symptoms and dysfunctional beliefs. Separate meta-regression analyses were also completed to examine the moderating role of age, sex, illness duration, negative symptom severity, and study quality, respectively. Given the variability in negative symptom scales used across studies, we applied a min-max normalization, resulting in a standardized scale of 0–10, with higher scores reflecting greater symptom severity. A minimum three-study threshold was used for all analyses.

*I^2^* and *τ^2^* statistics are reported as metrics of between-study heterogeneity. Several leave-one-out diagnostics (e.g. externally standardized residual, Cook’s distance, and covariance ratio) were computed using the ‘influence’ function in R to identify influential outlier studies. Publication bias was evaluated using Rosenthal’s *fail-safe N* and visual inspection of funnel plots.

## Results

### Selection of studies

**Figure 1.**
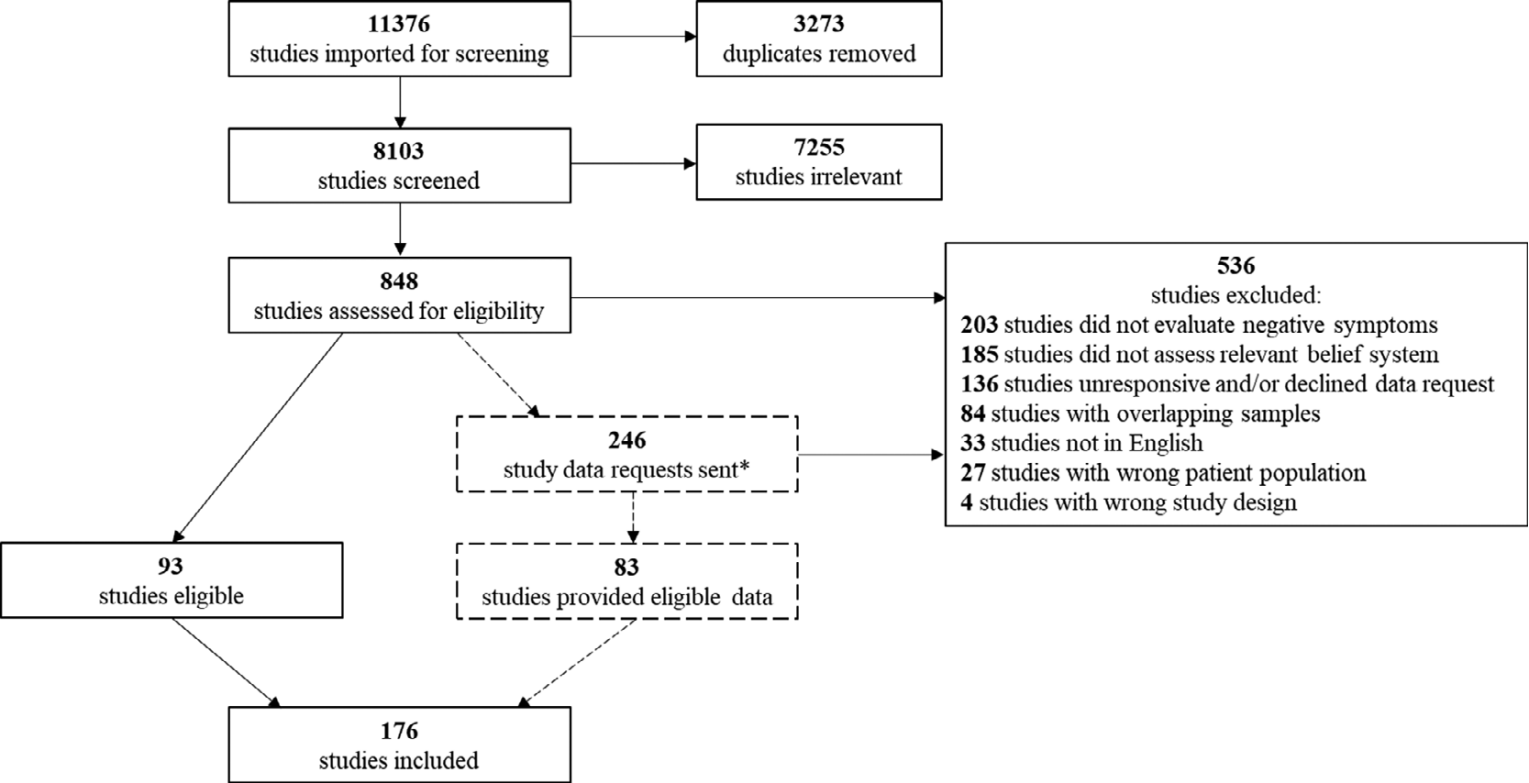
Flowchart illustrating the process of screening and selecting studies for meta-analysis. *Note: Reflects the number of studies only for which effect size data was requested, and without which the study would not be eligible for inclusion.

The PRISMA flowchart is illustrated in Figure 1. A total of 176 studies were included across meta-analyses. Study sample characteristics are summarized in Table 1 and fully described in the supplement.

**Table 1.** Summary of sample characteristics for studies included in the primary meta-analysis for each dysfunctional belief system

	*k* (# of Studies)	*n* (# of Participants)	Age *M*	% Male	Years of Illness *M*	Negative symptom severity (0–10) *M*	Study quality score (0–12) *M*
Defeatist performance beliefs	38	2808	41.2 (*k* = 37)	64.8% (*k* = 37)	17.2 (*k* = 24)	3.5 (*k* = 32)	9.1
Asocial beliefs	8	578	43.0	60.7%	23.2 (*k* = 5)	3.7	8.9
Low expectancies for success	55	5664	37.6	65%	12.8 (*k* = 43)	2.9 (*k* = 46)	9.1
Low expectancies for pleasure	5	249	35.0	66.4%	12.5 (*k* = 3)	2.7 (*k* = 4)	8.6
Internalized stigma	81	9766	38.4 (*k* = 79)	62.3% (*k* = 79)	13.9 (*k* = 63)	2.6 (*k* = 74)	9.1
Perception of limited resources	10	463	38.8	66%	21.4	3.2 (*k* = 9)	7.5

### Meta-analyses

**Figure 2.**
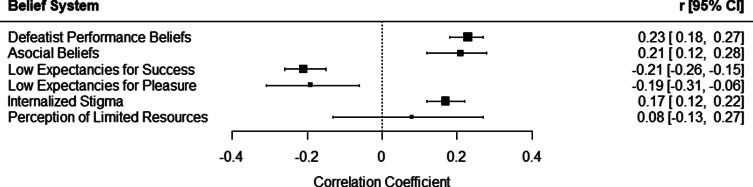
Summary of pooled results for primary random-effects meta-analyses evaluating relationships between overall negative symptoms and dysfunctional belief systems. An association of more severe negative symptoms with greater dysfunctional beliefs is represented by positive effect sizes for defeatist performance beliefs, asocial beliefs, internalized stigma, and perception of limited resources, and negative effect sizes for low expectancies for success and low expectancies for pleasure to reflect more negative (i.e. lower) expectations.

A summary of the primary pooled effects is illustrated in Figure 2. Subset analyses for all measures included in each primary meta-analysis, with *k* ≥ 3, are presented in Table 2. A full description of belief measures is presented in Table S1.

**Table 2.** Summary of subset analyses for different measures used to evaluate dysfunctional beliefs and negative systems, with *k* ≥ 3, in each primary meta-analysis

	*k*	*n*	*r*	95% CI	*p*	*I*^2^
Defeatist performance beliefs						
*Belief measures*						
DAS – DPB subscale	36	2741	0.23	0.18–0.27	<0.0001	27.8%
*Negative symptom measures*						
BNSS	11	682	0.24	0.16–0.32	<0.0001	15.8%
CAINS	5	366	0.23	0.13–0.33	<0.0001	0%
PANSS	7	345	0.25	0.15–0.35	<0.0001	0%
SANS	14	1384	0.22	0.13–0.30	<0.0001	59%
Asocial beliefs						
*Belief measures*						
ABS	6	511	0.20	0.11–0.28	<0.0001	0%
*Negative symptom measures*						
SANS	4	334	0.17	0.07–0.28	0.002	0%
Low expectancies for success						
*Belief measures*						
ES – self-efficacy	3	262	−0.15	−0.03 – −0.27	0.02	0.02%
GSES	12	1632	−0.13	−0.05 – −0.21	0.001	54.9%
PCS	5	244	−0.09	0.11 – −0.28	0.4	53.9%
RAS – success and goal orientation	13	172	−0.25	−0.14 – −0.35	<0.0001	72.3%
RSA – personal strength	4	1288	−0.10	0.02 – −0.21	0.09	58%
RSES	9	594	−0.29	−0.17 – −0.39	<0.0001	51.6%
*Negative symptom measures*						
BNSS	3	975	−0.18	−0.12 – −0.24	<0.0001	0%
BPRS	7	631	−0.13	0.06 – −0.31	0.2	80.1%
PANSS	34	3074	−0.17	−0.10 – −0.23	<0.0001	65.7%
SANS	9	818	−0.36	−0.26 – −0.46	<0.0001	52.1%
Low expectancies for pleasure						
*Belief measures*						
N/A*						
*Negative symptom measures*						
SANS	3	174	−0.13	0.02 – −0.28	0.08	0.01%
Internalized stigma						
*Belief measures*						
ISMI	67	8270	0.19	0.13–0.25	<0.0001	85.8%
PBIQ – entrapment/expectations	3	131	0.24	0.07–0.40	0.007	0%
SSS-S	5	766	0.07	−0.14 – 0.27	0.5	87%
*Negative symptom measures*						
BNSS	4	1099	0.22	0.16–0.27	<0.0001	0%
BPRS	8	686	0.19	0.10–0.27	<0.0001	17.3%
PANSS	48	5992	0.15	0.07–0.23	0.0002	88.5%
SANS	10	850	0.19	0.06–0.32	0.006	73.2%
Perception of limited resources						
*Belief measures*						
MCQ – cognitive confidence	7	250	−0.07	−0.30 – 0.16	0.5	62.0%
SARA-Q	3	213	0.31	0.17–0.42	<0.0001	0.01%
*Negative symptom measures*						
PANSS	5	185	−0.07	−0.34 – 0.22	0.7	61.2%

Abbreviations: ABS: Asocial Beliefs Scale; BNSS: Brief Negative Symptom Scale; BPRS: Brief Psychiatric Rating Scale; CAINS: Clinical Assessment Interview for Negative Symptoms; DAS-DPB: Dysfunctional Attitudes Scale – Defeatist Performance Beliefs; ES: Empowerment Scale – Self-Efficacy; GSES: General Self-Efficacy Scale; ISMI: Internalized Stigma of Mental Illness Scale; MCQ: Metacognitions Questionnaire – Cognitive Confidence; PANSS: Positive and Negative Syndrome Scale; Personal Beliefs about Illness Questionnaire – Entrapment/Expectations; PCS: Perceived Competency Scale; SANS: Scale for the Assessment of Negative Symptoms; RAS: Recovery Assessment Scale – Success and Goal Orientation; RSA: Resilience Scale for Adults – Personal Strength; RSES: Revised Self-Efficacy Scale; SARA-Q: Success and Resources Appraisals Questionnaire; SSS-S: Self-Stigma Scale – Short.

* *Note:* k < 3 for all measures of low expectancies for pleasure.

## Defeatist performance beliefs

### Study characteristics

Forty studies were included in the meta-analysis evaluating the relationship between negative symptoms and defeatist performance beliefs (Beck et al., 2013; Bennett, Brown, Fang, & Blanchard, 2023; Berry & Greenwood, 2018; Buchanan et al., 2021; Clay, Raugh, Bartolomeo, & Strauss, 2020; Couture, Blanchard, & Bennett, 2011; Décombe et al., 2021; Ebrahimi et al., 2021; Fisher et al., 2023; Granholm et al., 2020; Granholm et al., 2022; Granholm, Holden, Dwyer, & Link, 2020; Granholm, Holden, Link, & McQuaid, 2014; Granholm, Holden, Link, McQuaid, & Jeste, 2013; Grant & Beck, 2009; Green et al., 2022; Green, Hellemann, Horan, Lee, & Wynn, 2012; Kiwanuka, Strauss, McMahon, & Gold, 2014; Lee & Yu, 2023; Lincoln et al., 2010; Luther et al., 2023; McGovern, Reddy, Reavis, & Green, 2020; Park, Bennett, Couture, & Blanchard, 2013; Paul, Strauss, Gates-Woodyatt, Barchard, & Allen, 2023; Pillny, Krkovic, & Lincoln, 2018; Pillny & Lincoln, 2016; Pos et al., 2019; Raugh & Strauss, 2024; Rector, 2004; Reddy et al., 2018; Romanowska & Best, 2023; Saperia et al., 2019; Shaheen & Amin, 2016; Staring, ter Huurne, & van der Gaag, 2013; Strauss & Gold, 2016; Strauss, Morra, Sullivan, & Gold, 2015; Takeda et al., 2019; Thonon, Levaux, Della Libera, & Larøi, 2020; Ventura et al., 2014; Zhang, James, & Strauss, 2023), with 38 studies used in the main analysis with global negative symptoms (Table S2) and 31 studies used in the subdomain analyses (Table S2.1).

The main analysis (*k* = 38) consisted of 2808 unique participants, most of whom were outpatients, and in approximately half of the studies were described as clinically stable. The majority of these participants were male (64.8%, *k* = 37), and the mean age was 41.2 years (*k* = 37). Two studies selected younger participants between the ages of 18–36 as part of their inclusion criteria (Berry & Greenwood, 2018; Pos et al., 2019), while another two studies only included older participants over the age of 45 (Granholm et al., 2013; Granholm, Holden, Dwyer, & Link, 2020). Duration of illness data was available for 24 studies (*M* = 17.2 years). While most of these publications reported on patients experiencing chronic illness, three studies investigated early episode psychosis (Berry & Greenwood, 2018; Pos et al., 2019; Ventura et al., 2014). With respect to negative symptom severity, data was obtained for 32 studies (*M* = 3.5 on a standardized scale from 0 to 10). Nine studies specifically recruited patients with negative symptoms, with three studies requiring at least mild severity (Buchanan et al., 2021; Pillny et al., 2018; Pos et al., 2019), five studies with moderate–severe cut-offs (Bennett et al., 2023; Ebrahimi et al., 2021; Granholm et al., 2022; Granholm, Holden, Dwyer, Mikhael, et al., 2020; Staring et al., 2013), and one study including a subgroup of patients with deficit syndrome (Beck et al., 2013). Additionally, five studies excluded participants if they were in the acute phase of illness (Buchanan et al., 2021; Ebrahimi et al., 2021; Lee & Yu, 2023) or experienced positive symptoms greater than moderate severity (Granholm et al., 2022; Granholm, Holden, Dwyer, Mikhael, et al., 2020). Only one study selected patients based on the severity of defeatist performance beliefs (Granholm, Holden, Dwyer, Mikhael, et al., 2020).

### Meta-Analysis

The random-effects meta-analysis for the relationship between overall negative symptoms and defeatist performance beliefs (*k* = 38, *n* = 2808) revealed a significant correlation, *r* = 0.23 (95% CI, 0.18–0.27, *Z* = 9.9, *p* < 0.0001; Figure S1). Heterogeneity across studies was relatively small (*I^2^* = 25.3%, *τ^2^* = 0.005) and there were no significant moderators (Table S4). Visual inspection of the funnel plot (Figure S2), as well as Rosenthal’s *fail-safe N* = 1702, suggests publication bias is unlikely. Subset analyses for negative symptom subdomains (Table S3) revealed significant effects for diminished motivation (*k* = 31, *n* = 2452), *r* = 0.19 (95% CI, 0.14–0.23, *Z* = 8.8, *p* < 0.0001) and diminished expression (*k* = 24, *n* = 2053), *r* = 0.19 (95% CI, 0.12–0.25, *Z* = 5.8, *p* < 0.0001), with substantially more heterogeneity observed across studies in the diminished expression analysis (*I^2^* = 45.3%, *τ^2^* = 0.01) compared to diminished motivation (*I^2^* = 4.3%, *τ^2^* = 0.0006).

## Asocial beliefs

### Study characteristics

Eight studies (Beck et al., 2013; Buchanan et al., 2021; Granholm et al., 2022; Granholm, Holden, Dwyer, Mikhael, et al., 2020; Grant & Beck, 2010; Le, Holden, Link, & Granholm, 2018; Pillny et al., 2018; Zhang et al., 2023) were included in the primary meta-analysis assessing the relationship between negative symptoms and asocial beliefs (Table S7), with five of these studies also being used for the subdomain analyses (Table S7.1). The 578 participants across the eight studies were all outpatients, primarily male (60.7%), with a mean age of 43 years, 23.2 years of illness (*k* = 5), and an average negative symptom severity score of 3.7. Five studies specifically recruited participants who endorsed negative symptoms (Beck et al., 2013; Buchanan et al., 2021; Granholm et al., 2022; Grant & Beck, 2010; Pillny et al., 2018).

### Meta-Analysis

The meta-analysis revealed a significant correlation between overall negative symptoms and asocial beliefs (*k* = 8, *n* = 578), *r* = 0.21 (95% CI, 0.12–0.28, *Z* = 4.9, *p* < 0.0001; Figure S4). There was no heterogeneity across studies (*I^2^* = 0%, *τ^2^* = 0) and no significant moderators (Table S9). Publication bias is unlikely based on the funnel plot’s relative symmetry (Figure S5), and Rosenthal’s *fail-safe N* = 53. Subdomain analyses (Table S8) revealed a significant effect for diminished motivation (*k* = 5, *n* = 215), *r* = 0.15 (95% CI, 0.02–0.28, *Z* = 2.2, *p* = 0.03), but not diminished expression (*k* = 4, *n* = 184), *r* = 0.09 (95% CI, −0.06 – 0.24, *Z* = 1.2, *p* = 0.2). No heterogeneity was observed for either subdomain.

## Low expectancies for success

### Study characteristics

Sixty studies in total examined the relationship between negative symptoms and low expectancies for success (Avery, Startup, & Calabria, 2009; Beaudette, Cruz, Lukachko, Roché, & Silverstein, 2020; Bentall et al., 2010; Best, Milanovic, Iftene, & Bowie, 2019; Caqueo-Urízar, Ponce-Correa, Semir-González, & Urzúa, 2022; Cardenas et al., 2013; Cavelti, Wirtz, Corrigan, & Vauth, 2017; Chang et al., 2015, 2017; Cheng, Nadin, Bohonis, Katt, & Dewa, 2023; Chino, Nemoto, Fujii, & Mizuno, 2009; Choi, Fiszdon, & Medalia, 2010; Choi, Saperstein, & Medalia, 2012; Chrostek, Grygiel, Anczewska, Wciórka, & Świtaj, 2016; Clari et al., 2022; Cowan, Lundin, Moe, & Breitborde, 2023; Fisher et al., 2023; Fiszdon, Kurtz, Choi, Bell, & Martino, 2016; Galliot et al., 2022; García-Mieres, Lysaker, & Leonhardt, 2022; González-Domínguez, González-Sanguino, & Muñoz, 2019; Gruber et al., 2020; Haugen, Stubberud, Ueland, Haug, & Øie, 2021; Hayward et al., 2009; Herpertz et al., 2022; Hill & Startup, 2013; Huddy, Drake, & Wykes, 2016; Izydorczyk, Sitnik-Warchulska, Kühn-Dymecka, & Lizińczyk, 2019; Keefe et al., 2012; Kinoshita, Hashimoto, Nishimura, & Yotsumoto, 2023; Kukla, Strasburger, Salyers, Rollins, & Lysaker, 2021; Kurtz, Olfson, & Rose, 2013; Laxmi, Sahoo, Grover, & Nehra, 2023; Lee et al., 2017, 2021; Li, Wu, & Chen, 2023; Lim, Li, Xie, Tan, & Lee, 2021; Luther et al., 2015; Lysaker, Clements, Wright, Evans, & Marks, 2001; Markiewicz & Dobrowolska, 2021; Melau et al., 2015; Morgades-Bamba, Fuster-Ruizdeapodaca, & Molero, 2019; Murphy et al., 2023; Norman, Windell, Lynch, & Manchanda, 2013; Pratt, Mueser, Smith, & Lu, 2005; Priebe et al., 2015; Raffard et al., 2014; Rossi et al., 2017; Santosh & Kundu, 2023; Şenormancı et al., 2021; Şenormanci, Güçlü, & Şenormanci, 2022; Song et al., 2013; Strauss et al., 2015; Thonon et al., 2020; Vaskinn, Ventura, Andreassen, Melle, & Sundet, 2015; Vauth, Kleim, Wirtz, & Corrigan, 2007; Ventura et al., 2014; Watanabe, Taniguchi, & Sugihara, 2022; Wciórka, Świtaj, & Anczewska, 2015; Wright et al., 2021). Fifty-five of these studies were used for the primary analysis for the relationship with overall negative symptoms (Table S12), and 14 were included in the subdomain analyses (Table S12.1).

Within the 55 studies included within the primary analysis were 5664 unique participants who were mostly male (65%), outpatients, and with a mean age of 37.6 years. Five studies exclusively included younger participants (Cheng et al., 2023; Chino et al., 2009; Melau et al., 2015; Murphy et al., 2023; Song et al., 2013), while another two studies only recruited older participants (Cardenas et al., 2013; Wright et al., 2021). The average duration of illness was 12.8 years (*k* = 43), with nine studies specifically selecting first/early episode patients (Chang et al., 2015; Cheng et al., 2023; Cowan et al., 2023; Laxmi et al., 2023; Melau et al., 2015; Murphy et al., 2023; Norman et al., 2013; Song et al., 2013; Ventura et al., 2014). The mean negative symptom severity score was 2.9 (*k* = 46). Samples were characterized as clinically stable in nearly half of the included studies.

### Meta-analysis

The meta-analysis (*k* = 55, *n* = 5664) revealed a significant inverse correlation, *r* = −0.21 (95% CI, −0.15 – −0.26, *Z* = −7.3, *p* < 0.0001; Figure S7), such that more severe overall negative symptoms were associated with lower expectancies for success. There was substantial heterogeneity across studies (*I^2^* = 74.2%, *τ^2^* = 0.03), with age (ß = 0.007, *p* = 0.03) and illness duration (ß = 0.01, *p* = 0.02) as significant moderators (Table S14). One outlier study was identified (Santosh & Kundu, 2023) (Figure S9); though results were mostly unchanged when excluded (Tables S17–S19), but with reduced heterogeneity (*I^2^* = 67.3%) and only non-significant trends for moderation (age (*p* = 0.052), duration of illness (*p* = 0.07)). Publication bias is unlikely based on the funnel plots (Figures S8, S10), and Rosenthal’s *fail-safe N* = 3936. Subdomain analyses (Table S13) revealed significant effects for diminished motivation (*k* = 14, *n* = 2297), *r* = −0.33 (95% CI, −0.22 – −0.42, *Z* = −5.9, *p* < 0.0001) and diminished expression (*k* = 9, *n* = 1898), *r* = −0.21 (95% CI, −0.06 – −0.34, *Z* = −2.8, *p* = 0.005). Substantial heterogeneity was observed for both diminished motivation (*I^2^* = 81.4%, *τ^2^* = 0.03) and diminished expression (*I^2^* = 85.7%, *τ^2^* = 0.04).

## Low expectancies for pleasure

### Study characteristics

Five studies investigated the relationship between negative symptoms and low expectancies for pleasure (Beck et al., 2013; Hartmann et al., 2015; Hu et al., 2022; Strauss et al., 2015; Yang et al., 2018). One study (Hartmann et al., 2015) evaluated this association only with the amotivation subdomain and not global negative symptoms, but it was nonetheless included in the primary analysis in an attempt to maximize power (see Table S21 for sensitivity analysis).

The five studies consisted of 249 primarily outpatient participants, who were mostly male (66.4%), with a mean age of 35 years, 12.5 years of illness (*k* = 3), and an average negative symptom severity score of 2.7 (*k* = 4). One study included a subset of participants who were classified as having deficit syndrome (Beck et al., 2013).

### Meta-analysis

A significant inverse correlation emerged (*k* = 5, *n* = 249), *r* = −0.19 (95% CI, −0.06 – −0.31, *Z* = −2.9, *p* = 0.003; Figure S11), such that more severe negative symptoms were associated with lower expectancies for pleasure. There was no heterogeneity across studies and no significant moderators (Table S22). One study emerged as an outlier (Beck et al., 2013) (Figure S13), and its exclusion led to a somewhat larger effect size (*r* = −0.23) (Tables S24–S25). Based on inspection of funnel plots (Figures S12, S14), Rosenthal’s *fail-safe N* = 13, and the small number of studies included, these results are potentially susceptible to publication bias. There was no effect for the diminished motivation subdomain analysis (*k* = 3, *n* = 110), *r* = −0.21, (95% CI, 0.10 – −0.48, *Z* = −1.3, *p* = 0.2; Table S21).

## Internalized stigma

### Study characteristics

The meta-analysis for the relationship between negative symptoms and internalized stigma included 83 studies in total (Acosta, Navarro, Cabrera, Ramallo-Fariña, & Martínez, 2020; Barlati et al., 2022; Berry & Greenwood, 2018; Campellone, Caponigro, & Kring, 2014; Capatina & Miclutia, 2018; Caqueo-Urízar et al., 2022, 2019; Chan et al., 2019; Chen et al., 2016; Chrostek et al., 2016; Chu et al., 2023; Clari et al., 2022; Cloutier et al., 2023; Degnan, Berry, Vaughan, Crossley, & Edge, 2022; DeLuca et al., 2021; DeTore et al., 2022; Fekih-Romdhane, Hajje, Haddad, Hallit, & Azar, 2023; Feldhaus et al., 2018; Firmin et al., 2019; González-Domínguez et al., 2019; Grover et al., 2017; Grover, Sahoo, Chakrabarti, & Avasthi, 2018; Gruber et al., 2020; Hill & Startup, 2013; Hofer et al., 2016, 2019; Horsselenberg, Busschbach, Aleman, & Pijnenborg, 2016; Huang, Liu, & Yang, 2023; Ipci et al., 2020; Jian et al., 2022; Karidi et al., 2014, 2015; Khalaf, Fathy, Ebrahim, & Samie, 2023; Koçak et al., 2022; Konsztowicz, Gelencser, Otis, Schmitz, & Lepage, 2021; Krzyzanowski, Agid, Goghari, & Remington, 2021; Laxmi et al., 2023; Li et al., 2017; Lien et al., 2018; Lim et al., 2021; Lo et al., 2022; Luciano et al., 2021; Lv, Wolf, & Wang, 2013; Lysaker, Davis, Warman, Strasburger, & Beattie, 2007; Lysaker, Roe, & Yanos, 2007; Ma et al., 2023; MacDougall, Vandermeer, & Norman, 2015; Margetić, Jakovljević, Ivanec, Margetić, & Tošić, 2010; Morgades-Bamba et al., 2019; Murphy et al., 2023; Nabors et al., 2014; Ng, Yu, & Leung, 2024; O’Connor, Yanos, & Firmin, 2018; Ordóñez-Camblor, Paino, Fonseca-Pedrero, & Pizarro-Ruiz, 2021; Park et al., 2013; Pérez-Aguado et al., 2024; Pishdadian et al., 2023; Pos et al., 2019; Prouteau, Roux, Destaillats, & Bergua, 2017; Reneses et al., 2020; Rossi et al., 2017; Schrank, Amering, Hay, Weber, & Sibitz, 2014; Schwarzbold et al., 2021; Sen, Nehra, & Grover, 2020; Shaheen & Amin, 2016; Shin, Joo, & Kim, 2016; Singh, Mattoo, & Grover, 2016; Singla, Avasthi, & Grover, 2020; Staring et al., 2013; Styła & Świtaj, 2024; Suman, Nehra, Sahoo, & Grover, 2023; Swanson et al., 2022; Świtaj, Grygiel, Anczewska, & Wciórka, 2014; Tao et al., 2022; Tu, Liu, & Huang, 2023; Villagonzalo et al., 2019; Vrbova, Prasko, Holubova, Slepecky, & Ociskova, 2018; White, Haddock, Haarmans, & Varese, 2023; White, McCleery, Gumley, & Mulholland, 2007; Yanos et al., 2012; Yanos, Roe, Markus, & Lysaker, 2008; Yıldız, Kiras, İncedere, & Abut, 2019; Zhang et al., 2019), with 81 of these studies used for the primary analysis with global negative symptoms (Table S26) and 23 studies included in the subdomain analyses (Table S26.1).

The 81 studies included in the main analysis consisted of 9766 participants. Most of the participants were male (62.9%, *k* = 79) and outpatients and were described as clinically stable in slightly less than half of the studies. The mean age of these participants was 38.4 years (*k* = 79), with six studies specifically recruiting young people (Chen et al., 2016; Cloutier et al., 2023; DeLuca et al., 2021; Khalaf et al., 2023; Murphy et al., 2023; Pos et al., 2019). The average duration of illness was 13.9 years (*k* = 63), with 11 studies evaluating primarily first/early episode psychosis patients (Berry & Greenwood, 2018; Chen et al., 2016; Chu et al., 2023; Cloutier et al., 2023; DeLuca et al., 2021; DeTore et al., 2022; MacDougall et al., 2015; Murphy et al., 2023; Ng et al., 2024; Pos et al., 2019; Sen et al., 2020), seven studies with minimum illness or treatment duration inclusion cut-offs (Fekih-Romdhane et al., 2023; Grover et al., 2017, 2018; Huang et al., 2023; Konsztowicz et al., 2021; Singh et al., 2016; Singla et al., 2020), and one study recruiting both early phase and prolonged illness patients (Firmin et al., 2019). The mean negative symptom severity score was 2.6 (*k* = 74), with two studies specifically selecting participants endorsing at least mild or greater severity of negative symptoms (Pos et al., 2019; Staring et al., 2013). Three studies excluded participants with acute or high levels of psychotic symptoms (González-Domínguez et al., 2019; Horsselenberg et al., 2016; Koçak et al., 2022), and seven studies classified their samples as being in some degree of symptomatic remission (Grover et al., 2017; Karidi et al., 2015; Krzyzanowski et al., 2021; Sen et al., 2020; Singh et al., 2016; Singla et al., 2020; Suman et al., 2023). Two studies also specifically selected participants with elevated internalized stigma scores as part of their inclusion criteria (González-Domínguez et al., 2019; Yanos et al., 2012), with one additional study including a subsample of patients with at least moderate to high levels of internalized stigma (O’Connor et al., 2018).

### Meta-analysis

The meta-analysis revealed a significant correlation between overall negative symptoms and internalized stigma (*k* = 81, *n* = 9766), *r* = 0.17 (95% CI, 0.12–0.22, *Z* = 6.5, *p* < 0.0001; Figure S15). Heterogeneity across studies was high (*I^2^* = 83.7%, *τ^2^* = 0.04), with a non-significant trend for moderation by study quality (Table S28). Excluding the two outlier studies (Khalaf et al., 2023; Shaheen & Amin, 2016) (Figure S17) resulted in reduced heterogeneity (*I^2^* = 64%, *τ^2^* = 0.02), and significant moderating effects of negative symptom severity (β = 0.04, *p* = 0.004) and study quality (β = 0.05, *p* = 0.02 (Tables S31–S33). Based on funnel plots (Figures S16, S18) and Rosenthal’s *fail-safe N* = 7399, publication bias is unlikely. Significant effects emerged for diminished motivation (*k* = 23, *n* = 3255), *r* = 0.21 (95% CI, 0.15–0.28, *Z* = 6.1, *p* < 0.0001), but only trend-level for diminished expression (*k* = 16, *n* = 2256), *r* = 0.08 (95% CI, −0.01 – 0.16, *Z* = 1.8, *p* = 0.07) (Table S27). There was substantial heterogeneity for both diminished motivation (*I^2^* = 68.9%, *τ^2^* = 0.02) and diminished expression (*I^2^* = 66.6%, *τ^2^* = 0.02).

## Perception of limited resources

### Study characteristics

A total of 11 studies were included for the meta-analysis examining the relationship between negative symptoms and perception of limited resources (Bennett et al., 2023; Bortolon et al., 2014; Bröcker et al., 2017; Brüne, Drommelschmidt, Krüger-Özgürdal, & Juckel, 2019; Couture et al., 2011; Gesraha, Shalaby, & Harfush, 2023; Minor et al., 2022; Moritz, Peters, Larøi, & Lincoln, 2010; Østefjells et al., 2015; Popolo et al., 2017; Strauss et al., 2015), with 10 studies included in the primary analysis (Table S34) and three in the subdomain analysis (Table S34.1).

Across the 10 studies were 463 unique participants, 66% of whom were male, with a mean age of 38.8 and negative symptom severity score of 3.2 (*k* = 9). Duration of illness was only available for three studies (*M* = 21.4 years), with two studies specifically recruiting first/early episode psychosis patients (Brüne et al., 2019; Østefjells et al., 2015).

### Meta-analysis

A non-significant correlation, with high levels of heterogeneity across studies, emerged between overall negative symptoms and perception of limited resources (*k* = 10, *n* = 463), *r* = 0.08 (95% CI, −0.13 – 0.27, *Z* = 0.7, *p* = 0.5, *I^2^* = 75%, *τ^2^* = 0.07; Figure S19). There was, however, a significant effect specifically for the diminished motivation subdomain analysis (*k* = 3, *n* = 266), *r* = 0.29 (95% CI, 0.18–0.40, *Z* = 4.8, *p* < 0.0001), with no heterogeneity observed (Table S35). The funnel plot is presented in Figure S20.

## Discussion

The theorized cognitive model has become an increasingly popular psychological framework for conceptualizing negative symptoms in SSDs. The present study sought to critically appraise the extant empirical evidence related to the model by way of the largest and most comprehensive meta-analysis to date.

### Defeatist performance beliefs

In line with an older meta-analysis by Campellone, Sanchez, and Kring (2016), our updated findings revealed a small, significant relationship between overall negative symptoms and defeatist performance beliefs. Interestingly, our subdomain analyses showed no differences in effect sizes between diminished motivation and diminished expression, which were also both smaller in magnitude than the relationship with global negative symptoms. This is somewhat surprising given the conceptually stronger link between overgeneralized negative beliefs about performing goal-directed activity and diminished motivation in particular (Couture et al., 2011), which may call into question the specificity of this relationship. Nonetheless, our results provide further support for the importance of addressing self-defeating cognitions as treatment targets for negative symptoms.

### Asocial beliefs

Asocial beliefs, or negative and/or apathetic attitudes towards social engagement and affiliation, have been hypothesized to represent key processes underlying reduced motivation for interpersonal relationships in SSDs (Grant & Beck, 2010; Rector et al., 2005). While only a few studies were identified, our results offer promising preliminary support for this hypothesis, with findings of a small, significant correlation between asocial beliefs and overall negative symptom severity, as well as diminished motivation more specifically. With asocial beliefs emerging as potential mechanisms of change in CBT trials for negative symptoms (Granholm, Holden, & Worley, 2018), further research into this belief system and its relationship with the asociality features of negative symptoms may provide important insights for psychosocial intervention.

### Low expectancies for success

In classic theories of motivation, self-efficacy beliefs (i.e. the extent to which an individual believes in their ability to succeed) are central to engagement in goal-directed activity (Bandura, 1977). To this end, low expectancies for success, or reduced self-efficacy, have been positioned within the cognitive model as a critical belief system underlying negative symptoms, particularly amotivation. According to our meta-analysis, there was indeed a significant relationship between expectancies for success and overall negative symptom severity, with an even more pronounced effect for diminished motivation. Further, this relationship was moderated by age and illness duration, which may highlight the importance of early intervention to address and protect self-efficacy beliefs.

### Low expectancies for pleasure

Few studies were identified that examined low expectancies for pleasure. While this may be surprising given the large body of research dedicated to anhedonia in SSDs, our literature review revealed that most studies purporting to examine beliefs about pleasure utilized the TEPS anticipatory pleasure subscale (Gard, Gard, Kring, & John, 2006), which does not assess the beliefs or expectancies one holds regarding their likelihood of experiencing pleasure in the future (i.e. pleasure anticipation) but rather appears to measure one’s experience of pleasure in-the-moment while anticipating future events (i.e. anticipatory pleasure). As a result, only five studies, with questionable psychometric properties, were included in our meta-analysis, which revealed a significant, but tenuous correlation between expectancies for pleasure and negative symptom severity. While promising, the lack of well-established measures for this cognition necessitates further research and development, along with further delineation between pleasure expectancies versus anticipatory pleasure.

### Internalized stigma

Also known as self-stigmatization, internalized stigma refers to a process by which affected individuals self-identify with negative beliefs about their mental illness, incorporate stigmatizing views of the illness into their self-concept, and accept low expectations for themselves and their future as a result (Corrigan & Rao, 2012). Accordingly, the cognitive model posits that certain maladaptive beliefs endorsed by patients with negative symptoms are driven by internalized stigma (Rector et al., 2005). The results of our meta-analysis align with a smaller one by Sarraf, Lepage, and Sauvé (2022) and lend further support to this hypothesis in finding a significant relationship between negative symptom severity and internalized stigma, though the effect was relatively small and characterized by high levels of heterogeneity. There was, however, a more pronounced relationship with diminished motivation specifically, suggesting a role for demoralization in patients’ withdrawal from goal-directed activity and aligning with the ‘why try’ model of self-stigma (Corrigan, Bink, Schmidt, Jones, & Rüsch, 2016).

### Perception of limited resources

According to Rector et al. (2005), patients endorse statements such as ‘It’s too much’ and ‘I can’t handle it’ when asked to perform different tasks, and it is this subjective account of limited internal resources that contributes to a pattern of disengagement, passivity, and avoidance. Withdrawal from effortful activities and goal-directed behavior is therefore hypothesized to be partly due to beliefs characterized by underestimation of abilities and available resources, exaggeration of limitations, and overestimation of personal costs of expending energy. In the present meta-analysis, we identified very few studies that utilized measures that mapped onto this construct, despite including measures of both perception of cognitive resources and physical and psychological resources. Further, the effect was non-significant, though the inclusion of apparently disparate constructs potentially obfuscated the results, with the non-significant effect seemingly driven by the MCQ (i.e. cognitive resources). Thus, the available evidence as it currently stands does not support the relationship between perception of limited resources and overall negative symptoms; however, there remains a need to better define and evaluate this belief system, especially as it is often observed in patients clinically.

### Implications and future directions

It is important to acknowledge that the magnitude of the effect sizes reported above was mostly modest, though this is not entirely surprising given the extensive methodological and sample variability across studies, as well as the multifaceted nature of negative symptoms themselves. Indeed, negative symptoms are inherently complex clinical phenomena that are likely best explained by a multitude of interacting mechanisms, at both the individual and environmental levels (Strauss, 2021), but with potentially only small additive effects when examined on their own. Furthermore, our findings reflect population effect sizes amongst a heterogeneous sample, and therefore, it is not necessarily that dysfunctional beliefs exert only small effects for all patients with negative symptoms, nor is it the case that the five significant beliefs identified in this meta-analysis each contribute equally for every patient with negative symptoms. Instead, a more clinically meaningful approach going forward would be to focus on identifying the specific subgroups of patients for whom dysfunctional beliefs do relate to their negative symptoms, and then further subtype them based on their underlying belief profile. This would then allow for a more personalized approach to treatment planning and delivery, and may also pave the way for the development of a modularized treatment for negative symptoms, similar to what has been done for obsessive-compulsive disorder (Steketee et al., 2011; Wilhelm et al., 2009), whereby separate self-contained therapeutic modules are created for each dysfunctional belief system and then selectively matched with the patient based on their individual presentation and case conceptualization. This could serve to optimize the effectiveness and efficiency of future CBT interventions for negative symptoms.

### Limitations

The current review only evaluated the cross-sectional associations between negative symptoms and dysfunctional beliefs. Accordingly, it is not possible to determine causality or speak to the direction of the reported relationships. As few studies to date have utilized longitudinal designs, the extent to which different dysfunctional beliefs precede and predict the emergence and/or worsening of negative symptoms remains unknown. We were also unable to systematically ascertain whether negative symptoms reported across studies were primary or secondary to other factors, such as depressive or positive symptoms, which could also contribute to dysfunctional beliefs. Some of the meta-analyses also exhibited high levels of heterogeneity, potentially attributable to the wide range of included instruments for each belief system with possibly different operationalizations. Finally, while each of the dysfunctional beliefs examined in this meta-analysis was proposed as separate processes, it is highly possible that overlap exists among them. Unfortunately, however, factor analyses are limited in this regard, with the only potentially relevant analysis in this population, to our knowledge, demonstrating a relatively clear separation between defeatist performance beliefs and asocial beliefs (Pillny et al., 2018). Across studies included in this meta-analysis (*k* = 176), there were comparatively few that simultaneously evaluated more than one belief system (*k* = 26), and even fewer with available intercorrelation data (*k* = 10), with results that ranged widely from *r*s = |0.009–0.71|.| It will therefore be important for future research to conduct more fulsome concurrent examinations in order to identify the factor structure of these dysfunctional beliefs and their interrelationships.

### Summary

This comprehensive meta-analysis provides strong support and validation for the cognitive model of negative symptoms. Significant cross-sectional correlations with overall negative symptoms emerged for defeatist performance beliefs, asocial beliefs, low expectancies for success, low expectancies for pleasure, and internalized stigma, but not perception of limited resources. Subdomain analyses further suggested that many of these relationships appeared to be linked to diminished motivation specifically, which is particularly noteworthy from a clinical perspective given its association with poor outcomes. Longitudinal investigations of these relationships, along with improved measurement strategies, are important next steps to further delineate how dysfunctional beliefs contribute to negative symptoms in SSDs. The identification of these belief systems as clinically meaningful treatment targets may afford opportunities for the development of more optimized and precision-based CBT interventions for this critically unmet therapeutic need.

## Supporting information

Saperia et al. supplementary material Saperia et al. supplementary material

## Supplementary material

The supplementary material for this article can be found at http://doi.org/10.1017/S0033291724003325.
