# Supplementary material for: The cognitive model of negative symptoms: a systematic review and meta-analysis of the dysfunctional belief systems associated with negative symptoms in schizophrenia spectrum disorders
Source: Psychol Med. 2025 Feb 5;55:e11. doi: 10.1017/S0033291724003325 (PMC11968129; doi:10.1017/S0033291724003325)
Supplement: Saperia et al. supplementary material [file S0033291724003325sup001.docx]

**Supplement**

**Methods**

*Search Strategy*

The following search terms were used:

(schizo* or psychosis or non affective psychosis)

OR

psychosis/ or exp schizophrenia/

AND

((cognitive model adj4 negative symptom*) or ((expect* or belief* or attitude* or perce*) adj4 (pleasure or success or acceptance or performance or skill or social affiliation or asocial or abilit* or stigma* or resource* or dysfunctional or maladaptive)) or (defeatist adj4 (performance or belief* or attitude*)) or (self efficacy or self esteem or self stigma or self agency or internalized stigma) or appraisal).

*Quality Assessment*

The quality of individual studies was rated based on selection bias (i.e., recruited sample representative of the target population), study design (i.e., presence of clear hypothesis), data collection methods (i.e., use of valid and reliable measures of dysfunctional beliefs), and missing data (i.e., reported and/or addressed). Given that we requested specific post-hoc analyses to be conducted from several studies as part of our extensive request for data, we opted to exclude quality items related to the appropriateness of study analyses.

**Table S1.** Summary and description of measures used to evaluate each dysfunctional belief system across all included studies.

| Measure | *k* | Description |
| --- | --- | --- |
| ***Defeatist Performance Beliefs*** | | |
| Dysfunctional Attitudes Scale (DAS) –  Defeatist Performance Beliefs (DPB) | 38 | Subscale measuring overgeneralized maladaptive beliefs and defeatist attitudes towards performing goal-directed tasks. |
| Weissman & Beck Version | 33 | Original subscale as published. |
| Cane Version | 3 | Factor score consisting of 12 items from original subscale and 3 other DAS scale items. See Campellone, Sanchez, & Kring (2016) for distinction between versions. |
| Blatt Version | 1 | Orthogonal DPB dimension derived from the standardized residual after regressing the DAS need-for-approval factor on DPB Cane factor scores. See Rector (2004) |
| 10-item version | 1 | Comprised of first 10 items of original DPB subscale. |
| Demotivating Beliefs Inventory (DBI) –  Self-Defeating Beliefs | 1 | Contains 15 items from the DAS, including 14 from the Cane version of the DPB subscale, as well as 6 items from the Rosenberg Self-Esteem Scale. |
| Dysfunctional Beliefs Scale (DBS) – DPB | 1 | Comprised of 3 items from the original DPB subscale. |
|  | | |
| ***Asocial Beliefs*** | | |
| Asocial Beliefs Scale (ABS) | 6 | Comprised of 15 items from the Revised Social Anhedonia Scale measuring aversive social beliefs. |
| DBI – Social Indifference Beliefs | 1 | Contains 9 items from the ABS, along with 4 items from the Social Self-Efficacy Scale and 3 items from the DAS. |
| DBS – Asocial Beliefs | 1 | Comprised of 3 items from the ABS. |
|  | | |
| ***Low Expectancies for Success*** | | |
| Attitude Questionnaire – Self-Efficacy | 1 | Generalized. Derived from Magaletta and Oliver (Magaletta & Oliver, 1999). |
| Cognitive Expectancy Scale –  Self-Efficacy^1^ | 1 | Specific: expectations of success on the Trail Making Test B. |
| Cognitive Self-Efficacy Scale | 1 | Specific: belief in one’s ability to use cognitive functions effectively to attain desired goals. |
| Empowerment Scale – Self-Efficacy | 3 | Generalized. Factor score representing self-efficacy and self-esteem dimension of empowerment. |
| General Self-Efficacy Scale | 14 | Generalized. Perception of self-efficacy for managing challenges in life. |
| Memory Self-Efficacy Questionnaire | 1 | Specific: expectations of success completing memory exercises. |
| Motivation State Questionnaire – Self-Efficacy^a^ | 1 | Specific: confidence in ability to improve various domains in life. |
| Perceived Competency Scale | 6 | Specific: perceived self-competency about completing and mastering study-specific learning tasks. |
| Performance Expectancy | 1 | Specific: performance expectations on Mental Simulation Task. |
| Recovery Assessment Scale –  Success and Goal Orientation | 13 | Generalized. Factor score representing beliefs and attitudes about personal success and goal attainment. |
| Resilience Scale for Adults –  Personal Strength | 4 | Generalized. Factor score representing self-efficacy dimension of resilience. |
| Revised Self-Efficacy Scale | 9 | Specific: confidence in successfully performing everyday functional tasks and engaging in social activities. Excluding positive symptom self-efficacy subscale, where possible. Developed specifically for SSD populations. |
| Self-Efficacy for Community Life Scale | 1 | Specific: self-efficacy for living in the community across domains of daily living, behaviour in relation to treatment, behaviour in relation to symptoms, social life, and interpersonal relation. Developed specifically for SSD populations. |
| Self-Efficacy Questionnaire | 1 | Specific: performance expectancies on the Faux Pas social cognition test. |
| Self-Efficacy Scale – General | 1 | Generalized. Factor score representing general self-efficacy expectancies and attitudes. |
| Word Memory Test – Success Expectancy | 1 | Specific: expectation of success on Green’s Word Memory Test. |
| Task Motivation Questionnaire^a^ | 1 | Specific: expectations of success completing various routine activities. |
|  | | |
| ***Low Expectancies for Pleasure*** | | |
| Low Expectation of Future Pleasure | 1 | Specific: expectations of pleasure for social activity, physical sensation, and recreational/vocational pursuits. |
| Anticipated Monetary Reward Pleasure | 1 | Specific: amount of pleasure expected to feel upon unexpectedly finding money on the street. |
| Word Memory Test – Expected Enjoyment | 1 | Specific: expectation of enjoyment on Green’s Word Memory Test. |
| Beliefs about Pleasure Scale | 2 | Generalized. Subscales include Devaluation of Pleasure, Pleasurable Activity Expectancies, Negative Outcome Expectancies, and Attention to Pleasure. |
|  | | |
| ***Internalized Stigma*** | | |
| Internalized Stigma of Mental Illness (ISMI) Scale | 67 | Endorsement of self-stigmatizing beliefs. Consists of 5 subscales: Alienation; Stereotype Endorsement; Perceived Discrimination; Social Withdrawal; and Stigma Resistance. |
| Excluding Stigma Resistance subscale | 7 | Total score excluding Stigma Resistance subscale. Based on findings of weak factor loading. |
| Alienation & Stereotype Endorsement subscales^a^ | 1 | Alienation and Stereotype Endorsement subscales only. |
| ISMI-9 | 1 | Brief 9-item version. |
| ISMI-10 | 2 | Brief 10-item version. |
| ISMI-12 | 1 | Brief 12-item version. |
| Personal Beliefs about Illness Questionnaire – Entrapment/Expectations | 3 | Subscale representing perceived defeat and inability to reaffirm identity or sense of belonging due to illness. |
| Self-Stigma Inventory for Patients with Schizophrenia | 1 | Comprised of 3 factors: perceived devaluation; internalized stereotypes and social withdrawal; and concealment of the illness. Developed specifically for SSD populations, while also in consideration of Turkish culture and stereotypes. |
| Self-Stigma Questionnaire | 1 | Comprised of 3 factors: social discrimination; perceived capabilities; and concealment of the disease. Developed specifically for SSD populations based on patient focus groups. |
| Self-Stigma Scale – Short Form | 5 | Shortened 9-item version. Evaluates cognitive, affective, and behavioural dimensions of self-stigmatization. |
| Self-Stigma of Mental Illness Scale | 2 | Comprised of 4 levels of self-stigmatization in mental illness: stereotype awareness; stereotype agreement; self-concurrence; and self-esteem decrement. |
| Stigma Inventory for Mental Illness –  Self-Image/Self-Efficacy | 2 | Factor score representing perceived self-image and self-efficacy due to mental illness. |
|  | | |
| ***Perception of Limited Resources*** | | |
| Metacognitions Questionnaire –  Cognitive Confidence | 8 | Perception of limited cognitive resources. Factor score representing reduced confidence in attention and memory. |
| Success and Resources Appraisals Questionnaire | 3 | Perception of limited psychological and physical resources (e.g., “I just don’t have the energy to do much”). Also incorporates questions regarding expectancies for success. |

^a^Only used in studies included in the subdomain analyses. Note: a distinction is made between generalized and domain-specific expectancies for success and pleasure.

**Results**

Supplementary results, along with the corresponding supplementary tables and figures, are presented below, organized by dysfunctional belief system.

**Defeatist Performance Beliefs**

**Table S2.** Summary of studies included in primary meta-analysis for defeatist performance beliefs.

| Study | *N* | Country | Diagnosis | Status | Illness Duration – Years *M* (SD) | Age *M* (SD) | % Male | Negative Symptom Severity | Negative Symptom Measure | DPB Measure |
| --- | --- | --- | --- | --- | --- | --- | --- | --- | --- | --- |
| Beck et al. (2013)^a^ | 94 | USA | SZ | Outpatients | 17.93 | 39.5 | 75.5% | 2.8 | PDS/BPRS | DAS-DPB |
| Bennett et al. (2023) | 105 | USA | SZ/SA | Outpatients | 25.32^b^ | 55.6 (10.6) | 91.4% | 4.8 | CAINS | DAS-DPB |
| Berry & Greenwood (2018) | 50^c^ | England | SSD+ | Outpatients | 2.99 (3.6)^b^ | 26.1 (5.6) | 58.8% | 1.6 | PANSS | DAS-DPB |
| Buchanan et al. (2021) | 62 | USA | SZ/SA | Outpatients |  | 41.8 | 61.3% | 3.7 | SANS | DAS-DPB |
| Clay et al. (2020) | 186 | USA | SZ/SA | Outpatients |  | 39.6 (11.2) | 60.8% |  | BNSS | DAS-DPB |
| Couture et al. (2011) | 62 | USA | SZ/SA | Outpatients | 24.9^d^ | 46.7 (8.4) | 62.9% | 3.2 | CAINS | DAS-DPB |
| Décombe et al. (2021) | 32 | France | SZ |  | 7.42 (7.2) | 30.6 (7.5) | 71.9% | 0.95 | PANSS | DAS-DPB |
| Ebrahimi et al. (2021) | 85 | Iran | SZ/SA |  | 13 (1.7) | 45.6 (9) | 100% | 4.3 | SANS | DAS-DPB |
| Granholm et al. (2013) | 64 | USA | SZ/SA | Outpatients | 30.4 (13.6) | 55 (6.6) | 55% | 4.0 | SANS | DAS-DPB |
| Granholm et al. (2014) | 149 | USA | SZ/SA | Outpatients | 21.3 | 41.35 | 66.4% | 4.0 | SANS | DAS-DPB |
| Granholm et al. (2020a) | 31 | USA | SZ/SA | Outpatients | 28.8 (11.4) | 48.3 (9.5) | 65% | 5.4 | CAINS | DAS-DPB |
| Granholm et al. (2020b) | 57 | USA | SZ/SA | Outpatients | 30.6 (12) | 55.9 | 82.5% | 3.9 | SANS | DAS-DPB |
| Granholm et al. (2022) | 55 | USA | SZ/SA | Outpatients | 30.3 (12.3) | 50.64 | 64.7% | 3.5 | SANS | DAS-DPB |
| Grant & Beck (2009) | 55 | USA | SZ/SA | Outpatients | 14.4 (9.8) | 36.9 (9.9) | 65% | 2.8 | SANS | DAS-DPB |
| Green et al. (2012) | 191 | USA | SZ/SA | Outpatients | 24.2 (11.3) | 46.6 (9.8) | 67.5% | 3.9 | SANS | DAS-DPB |
| Green et al. (2022) | 75^e^ | USA | SSD |  |  | 48.7 (11) | 96.3% | 4.7 | CAINS | DAS-DPB |
| Kiwanuka et al. (2014) | 100 | USA | SZ/SA | Outpatients |  | 41.1 (10.6) | 68% | 2.9 | SANS | DAS-DPB |
| Lee & Yu (2023) | 331 | Taiwan | SZ | Outpatients |  | 55.6 (7.3) | 71.6% | 4.2 | SANS^f^ | DAS-DPB |
| Lincoln et al. (2010) | 100^g^ | Germany | SSD | Mixed (O) | 11 (9.1) | 34.1 (11.8) | 66.6% |  | PANSS | DAS-DPB |
| Luther et al. (2023) | 30^h^ | USA | SZ/SA | Outpatients |  | 43.4 (9.7) | 48.1% | 2.7 | BNSS | DAS-DPB |
| Park et al. (2013)^i^ | 49 | USA | SZ/SA | Outpatients |  | 49.6 (7.2) | 71.4% |  | SANS | DAS-DPB |
| Paul et al. 2023 | 101 | USA | SZ/SA | Outpatients |  |  |  |  | BNSS | DAS-DPB |
| Pillny & Lincoln (2016) | 58 | Germany | SSD | Outpatients | 12.1 (8) | 35.7 (12.7) | 60.3% | 4.5 | BNSS | DAS-DPB |
| Pillny et al. (2018) | 36 | Germany | SZ/SA | Outpatients | 17.7 (11.3)^j^ | 44.2 (10.9) | 56% | 3.9 | BNSS | DBI-SDB |
| Pos et al. (2019) | 75^k^ | Netherlands | SSD | Mixed | < 4 | 25.43 | 80.8% | 3.7 | BNSS | DAS-DPB |
| Raugh & Strauss (2024) | 42 | USA | SZ/SA | Outpatients |  | 39.5 (12.9) | 37.2% | 1.9 | BNSS | DAS-DPB |
| Rector (2004) | 56 | Canada | SZ/SA | Outpatients | 17 | 37.7 (9.3) | 41% |  | PANSS | DAS-DPB |
| Reddy et al. (2018) | 93^l^ | USA | SZ | Outpatients | 26.4 | 48.8 (11.6) | 51.2% | 4.0 | CAINS | DAS-DPB |
| Romanowska & Best (2023) | 30 | Canada | SSD | Outpatients |  | 29.2 (10.3) | 41.4% | 1.6 | BNSS | DAS-DPB^m^ |
| Saperia et al. (2019) | 21 | Canada | SZ/SA | Outpatients | 9.2 (6.3) | 31.5 (8.7) | 47.6% | 3.5 | SANS | DAS-DPB |
| Shaheen & Amin (2016) | 50 | Pakistan | SZ/SA |  | 7 (4.3) | 38 (7.3) | 68% | 3.3 | PANSS | DAS-DPB |
| Staring et al. (2013) | 21 | Netherlands | SSD | Outpatients | 13 | 40.6 | 66.7% | 3.1 | PANSS | DAS-DPB |
| Strauss et al. (2015) | 46 | USA | SZ/SA | Outpatients |  | 41.7 (10.7) | 60.9% |  | BNSS | DAS-DPB |
| Strauss & Gold (2016) | 60^n^ | USA | SZ/SA | Outpatients |  | 40.1 (11.2) | 73.8% | 3.1 | BNSS | DAS-DPB |
| Takeda et al. (2019) | 36 | Japan | SZ |  | 16 (7.12) | 42.9 (9.4) | 44.4% | 2.0 | PANSS | DAS-DPB |
| Thonon et al. (2020) | 18 | Belgium | SZ/SA | Mixed (O) | 11.1 (6) | 36.3 (8.2) | 89% | 5.1 | BNSS | DAS-DPB |
| Ventura et al. (2014) | 71 | USA | SSD | Outpatients | 0.5 (.5) | 21.7 (3.3) | 80% | 4.0 | SANS | DAS-DPB |
| Zhang et al. (2023) | 31 | USA | SZ | Outpatients |  | 39.6 (13.4) | 29% | 4.9 | NSI-SR | DBS-DPB^o^ |

Abbreviations: BNSS: Brief Negative Symptom Scale; BPRS: Brief Psychiatric Rating Scale; CAINS: Clinical Assessment Interview for Negative Symptoms; DAS-DPB: Dysfunctional Attitudes Scale – Defeatist Performance Beliefs; DBS-DPB: Dysfunctional Beliefs Scale – Defeatist Performance Beliefs; DBI-SDB: Demotivating Beliefs Inventory – Self-Defeating Beliefs; Mixed (O): Majority Outpatients; NSI-SR: Negative Symptom Inventory – Self-Report; PANSS: Positive and Negative Syndrome Scale; PDS: Proxy for the Deficit Syndrome; SA: Schizoaffective Disorder; SANS: Scale for the Assessment of Negative Symptoms; SSD: Schizophrenia-Spectrum Disorder; SSD+: Schizophrenia-Spectrum Disorder + Other Diagnoses (≤ 15%); SZ: Schizophrenia.

^a^Effect size (*r*) calculated using adjusted means and standard errors from comparing difference in DPB between high and low deficit syndrome participants, while controlling for depression.

^b^Reflects length of mental health service use

^c^N=51 for demographic information.

^d^Calculated by subtracting mean age of first psychiatric treatment from mean age.

^e^N=80 for demographic information.

^f^Revised total score that excludes SANS alogia and attention impairment subscales.

^g^N=83 for demographic information.

^h^N=27 for demographic information.

^i^Effect size (*r* = 0.004) obtained from Campellone et al. (2016) meta-analysis.

^j^Obtained from Pillny, Schlier, & Lincoln, (2020), N=35.

^k^N=99 for demographic information.

^l^N=94 for demographic information (except duration of illness, N=72).

^m^Comprised of first 10 scale items only.

^n^N=65 for demographic information.

^o^Subscale is comprised of 3 items from the DAS-DPB.

| Study | N | Diagnosis | Diminished Motivation Measure | Diminished Expression Measure | DPB Measure |
| --- | --- | --- | --- | --- | --- |
| Bennett et al. (2023) | 105 | SZ/SA | CAINS-MAP |  | DAS-DPB |
| Buchanan et al. (2021) | 62 | SZ/SA | SANS Experiential | SANS Expressive | DAS-DPB |
| Clay et al. (2020) | 186 | SZ/SA | BNSS-MAP | BNSS-EXP | DAS-DPB |
| Couture et al. (2011) | 62 | SZ/SA | CAINS Diminished Experience | CAINS Diminished Expression | DAS-DPB |
| Décombe et al. (2021) | 32 | SZ | PANSS Amotivation |  | DAS-DPB |
| Ebrahimi et al. (2021) | 85 | SZ/SA | SANS Diminished Motivation | SANS Diminished Expression | DAS-DPB |
| Fisher et al. (2023) | 100 | SSD | MAP-SR |  | DAS-DPB |
| Granholm et al. (2013) | 64 | SZ/SA | SANS Diminished Motivation | SANS Diminished Expression | DAS-DPB |
| Granholm et al. (2014) | 149 | SZ/SA | SANS Diminished Motivation | SANS Diminished Expression | DAS-DPB |
| Granholm et al. (2020a) | 31 | SZ/SA | CAINS-MAP | CAINS-EXP | DAS-DPB |
| Granholm et al. (2020b) | 57 | SZ/SA | SANS Diminished Motivation | SANS Diminished Expression | DAS-DPB |
| Granholm et al. (2022) | 55 | SZ/SA | SANS Diminished Motivation | SANS Diminished Expression | DAS-DPB |
| Green et al. (2012) | 191 | SZ/SA | SANS Experiential | SANS Expressive | DAS-DPB |
| Green et al. (2022) | 41 | SSD+ | CAINS-MAP | CAINS-EXP | DAS-DPB |
| Kiwanuka et al. (2014) | 100 | SZ/SA | SANS Diminished Motivation | SANS Diminished Expression | DAS-DPB |
| Lee & Yu (2023) | 331 | SZ | SANS Diminished Motivation | SANS Diminished Expression | DAS-DPB |
| Luther et al. (2023) | 30 | SZ/SA | BNSS-MAP | BNSS-EXP | DAS-DPB |
| McGovern et al. (2020) | 66 | SZ/SA | CAINS-MAP |  | DAS-DPB |
| Pillny & Lincoln (2016) | 58 | SSD | BNSS-MAP | BNSS-EXP | DAS-DPB |
| Pillny et al. (2018) | 36 | SZ/SA | BNSS-MAP | BNSS-EXP | DBI-SDB |
| Pos et al. (2019) | 75 | SSD | BNSS-MAP | BNSS Blunted Affect | DAS-DPB |
| Raugh & Strauss (2024) | 42 | SZ/SA | BNSS-MAP | BNSS-EXP | DAS-DPB |
| Reddy et al. (2018) | 93 | SZ | CAINS-MAP | CAINS-EXP | DAS-DPB |
| Romanowska & Best (2023) | 30 | SSD | MAP-SR |  | DAS-DPB^a^ |
| Saperia et al. (2019) | 21 | SZ/SA | SANS Diminished Motivation | SANS Diminished Expression | DAS-DPB |
| Strauss & Gold (2016) | 60 | SZ/SA | BNSS-MAP | BNSS-EXP | DAS-DPB |
| Takeda et al. (2019) | 36 | SZ | QLS Intrapsychic Foundations |  | DAS-DPB |
| Thonon et al. (2020) | 18 | SZ/SA | BNSS-MAP | BNSS-EXP | DAS-DPB |
| Ventura et al. (2014) | 71 | SSD | SANS Experiential | SANS Expressive | DAS-DPB |
| Zhang et al. (2023) | 31 | SZ | NSI-SR Diminished Motivation |  | DBS-DPB^b^ |

**Table S2.1.** Summary of studies included in subdomain analyses for defeatist performance beliefs.

Abbreviations: BNSS: Brief Negative Symptom Scale; CAINS: Clinical Assessment Interview for Negative Symptoms; DAS-DPB: Dysfunctional Attitudes Scale – Defeatist Performance Beliefs; DBI-SDB: Demotivating Beliefs Inventory – Self-Defeating Beliefs; DBS-DPB: Dysfunctional Beliefs Scale – Defeatist Performance Beliefs; EXP: Expressivity subscale; MAP: Motivation and Pleasure subscale; MAP-SR: Motivation and Pleasure Scale – Self-Report; NSI-SR: Negative Symptom Inventory – Self-Report; PANSS: Positive and Negative Syndrome Scale; QLS: Quality of Life Scale; SA: Schizoaffective Disorder; SANS: Scale for the Assessment of Negative Symptoms; SSD: Schizophrenia-Spectrum Disorder; SSD+: Schizophrenia-Spectrum Disorder + Other Diagnoses (≤ 15%); SZ: Schizophrenia.

^a^Comprised of first 10 scale items only.

^b^Subscale is comprised of 3 items from the DAS-DPB.

**
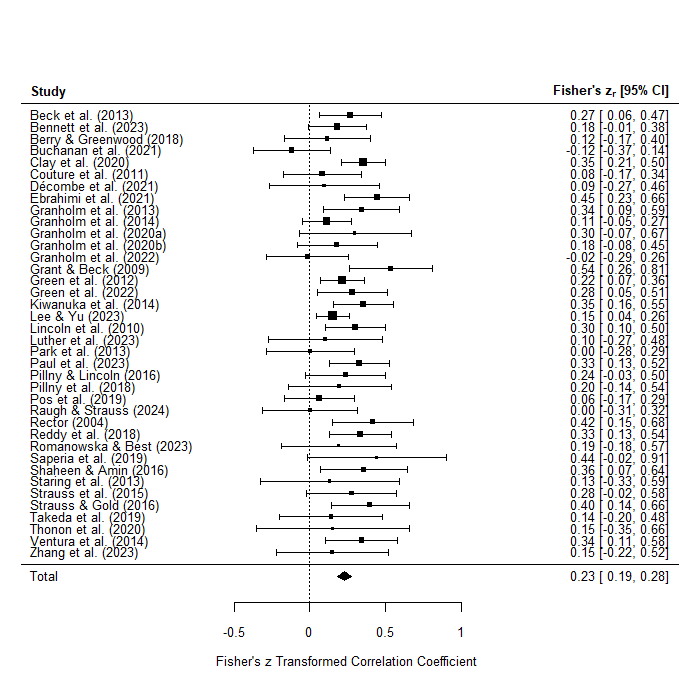
**

**Figure S1.** Forest plot of studies included in primary meta-analysis between defeatist performance beliefs and overall negative symptoms. Effect sizes represented by *z*-transformed Pearson’s *r* correlations.

**Table S3.** Meta-analysis for relationship between defeatist performance beliefs and total negative symptoms, as well as subset analyses for negative symptom subdomains.

|  | Number of Studies (*k*) | Number of Participants (*n*) | Effect Size (*r*) | 95% CI | *p* | SE | *Z* | *I^2^* | *τ^2^* |
| --- | --- | --- | --- | --- | --- | --- | --- | --- | --- |
| Total Negative Symptoms | 38 | 2808 | 0.23 | 0.18 – 0.27 | <0.0001 | 0.02 | 9.9 | 25.3% | 0.005 |
| Diminished Motivation Subdomain | 31 | 2452 | 0.19 | 0.14 – 0.23 | <0.0001 | 0.02 | 8.8 | 4.3% | 0.0006 |
| Diminished Expression Subdomain | 24 | 2053 | 0.19 | 0.12 – 0.25 | <0.0001 | 0.03 | 5.8 | 45.3% | 0.01 |

**Table S4.** Meta-regression analyses of moderators of relationship between overall negative symptoms and defeatist performance beliefs.

|  | Number of Studies (*k*) | Number of Participants (*n*) | ß | SE | *p* |
| --- | --- | --- | --- | --- | --- |
| Age | 37 | 2707 | -0.002 | 0.003 | 0.5 |
| Sex (% Male) | 37 | 2707 | 0.001 | 0.002 | 0.5 |
| Years of Illness | 24 | 1590 | -0.005 | 0.003 | 0.1 |
| Negative Symptom Severity | 32 | 2270 | 0.007 | 0.03 | 0.8 |
| Study Quality | 38 | 2808 | 0.005 | 0.03 | 0.9 |

**Table S5.** Subset analyses for each measure of defeatist performance beliefs (minimum *k* = 3) included in primary meta-analysis.

|  | Number of Studies (*k*) | Number of Participants (*n*) | Effect Size (*r*) | 95% CI | *p* | SE | *Z* | *I^2^* | *τ^2^* |
| --- | --- | --- | --- | --- | --- | --- | --- | --- | --- |
| DAS-DPB | 36 | 2741 | 0.23 | 0.18 – 0.27 | <0.0001 | 0.02 | 9.6 | 27.8% | 0.005 |
| Weissman & Beck Version | 31 | 2411 | 0.23 | 0.18 – 0.27 | <0.0001 | 0.03 | 8.8 | 29.9% | 0.006 |
| Cane Version | 3 | 244 | 0.21 | 0.04 – 0.37 | 0.01 | 0.09 | 2.5 | 34.05% | 0.008 |

Abbreviations: DAS-DPB: Dysfunctional Attitudes Scale – Defeatist Performance Beliefs

**Table S6.** Subset analyses for each negative symptom measure (minimum *k* = 3) included in primary meta-analysis for defeatist performance beliefs.

|  | Number of Studies (*k*) | Number of Participants (*n*) | Effect Size (*r*) | 95% CI | *p* | SE | *Z* | *I^2^* | *τ^2^* |
| --- | --- | --- | --- | --- | --- | --- | --- | --- | --- |
| BNSS | 11 | 682 | 0.24 | 0.16 – 0.32 | <0.0001 | 0.04 | 5.5 | 15.8% | 0.003 |
| CAINS | 5 | 366 | 0.23 | 0.13 – 0.33 | <0.0001 | 0.05 | 4.4 | 0% | 0 |
| PANSS | 7 | 345 | 0.25 | 0.15 – 0.35 | <0.0001 | 0.06 | 4.0 | 0% | 0 |
| SANS | 14 | 1384 | 0.22 | 0.13 – 0.30 | <0.0001 | 0.05 | 4.9 | 59% | 0.02 |

Abbreviations: BNSS: Brief Negative Symptom Scale; CAINS: Clinical Assessment Interview for Negative Symptoms; PANSS: Positive and Negative Syndrome Scale; SANS: Scale for the Assessment of Negative Symptoms.


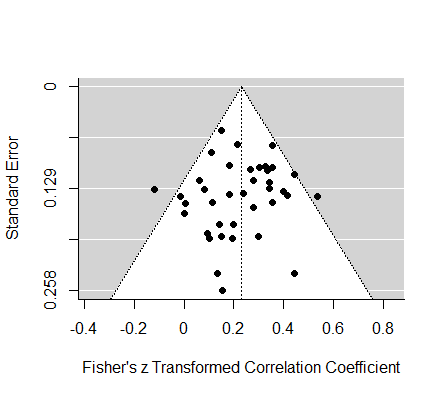


**Figure S2.** Funnel plot for primary meta-analysis of defeatist performance beliefs.


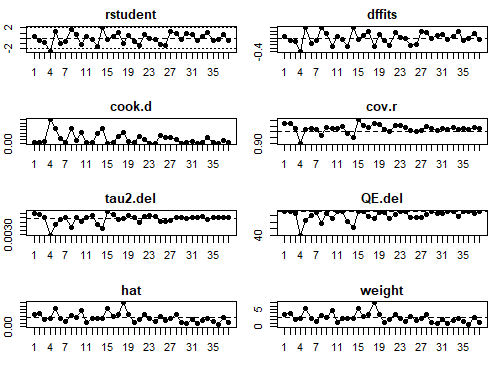


**Figure S3.** Influential outlier analyses for studies included in the primary meta-analysis for defeatist performance beliefs (*k* = 38). Significant studies are illustrated by the presence of red dots.

**Asocial Beliefs**

**Table S7.** Summary of studies included in primary meta-analysis for asocial beliefs.

| Study | N | Country | Diagnosis | Status | Illness Duration – Years *M* (SD) | Age *M* (SD) | % Male | Negative Symptom Severity | Negative Symptom Measure | Asocial Beliefs Measure |
| --- | --- | --- | --- | --- | --- | --- | --- | --- | --- | --- |
| Beck et al. (2013)^a^ | 94 | USA | SZ | Outpatients | 17.93 | 39.5 | 75.5% | 2.8 | SANS | ABS |
| Buchanan et al. (2021) | 62 | USA | SZ/SA | Outpatients |  | 41.8 | 61.3% | 3.7 | SANS | ABS |
| Granholm et al. (2020a) | 31 | USA | SZ/SA | Outpatients | 28.8 (11.4) | 48.3 (9.5) | 65% | 5.4 | CAINS | ABS |
| Granholm et al. (2022) | 55 | USA | SZ/SA | Outpatients | 30.3 (12.3) | 50.64 | 64.7% | 3.5 | SANS | ABS |
| Grant & Beck (2010) | 123 | USA | SZ/SA | Outpatients |  | 38.6 (12.1) | 65.8% | 3.2 | SANS | ABS |
| Le et al. (2018) | 146 | USA | SZ/SA | Outpatients | 21.3 (10.9) | 41.5 (9.6) | 68% | 2.0 | PANSS | ABS |
| Pillny et al. (2018) | 36 | Germany | SZ/SA | Outpatients | 17.7 (11.3)^b^ | 44.2 (10.9) | 56% | 3.9 | BNSS | DBI-SIB |
| Zhang et al. (2023) | 31 | USA | SZ | Outpatients |  | 39.6 (13.4) | 29% | 4.9 | NSI-SR | DBS-ASB |

Abbreviations: ABS: Asocial Beliefs Scale; BNSS: Brief Negative Symptom Scale; CAINS: Clinical Assessment Interview for Negative Symptoms; DBI-SIB: Demotivating Beliefs Inventory – Social Indifference Beliefs; DBS-ASB: Dysfunctional Beliefs Scale – Asocial Beliefs; PANSS: Positive and Negative Syndrome Scale; NSI-SR: Negative Symptom Inventory – Self-Report; SA: Schizoaffective Disorder; SANS: Scale for the Assessment of Negative Symptoms; SZ: Schizophrenia.

^a^Effect size (*r*) calculated using adjusted means and standard errors from comparing difference in ABS between high and low deficit syndrome participants, while controlling for depression.

^b^Obtained from Pillny, Schlier, & Lincoln (2020), N=35

**Table S7.1.** Summary of studies included in subdomain analyses for asocial beliefs.

| Study | N | Diagnosis | Diminished Motivation Measure | Diminished Expression Measure | Asocial Beliefs Measure |
| --- | --- | --- | --- | --- | --- |
| Buchanan et al. (2021) | 62 | SZ/SA | SANS Experiential | SANS Expressive | ABS |
| Granholm et al. (2020a) | 31 | SZ/SA | CAINS-MAP | CAINS-EXP | ABS |
| Granholm et al. (2022) | 55 | SZ/SA | SANS Diminished Motivation | SANS Diminished Expression | ABS |
| Pillny et al. (2018) | 36 | SZ/SA | BNSS-MAP | BNSS-EXP | DBI-SIB |
| Zhang et al. (2023) | 31 | SZ | NSI-SR Diminished Motivation |  | DBS-ASB |

Abbreviations: ABS: Asocial Beliefs Scale; BNSS: Brief Negative Symptom Scale; CAINS: Clinical Assessment Interview for Negative Symptoms; DBI-SIB: Demotivating Beliefs Inventory – Social Indifference Beliefs; DBS-ASB: Dysfunctional Beliefs Scale – Asocial Beliefs; EXP: Expressivity subscale; MAP: Motivation and Pleasure subscale; NSI-SR: Negative Symptom Inventory – Self-Report; SA: Schizoaffective Disorder; SANS: Scale for the Assessment of Negative Symptoms; SZ: Schizophrenia.

*
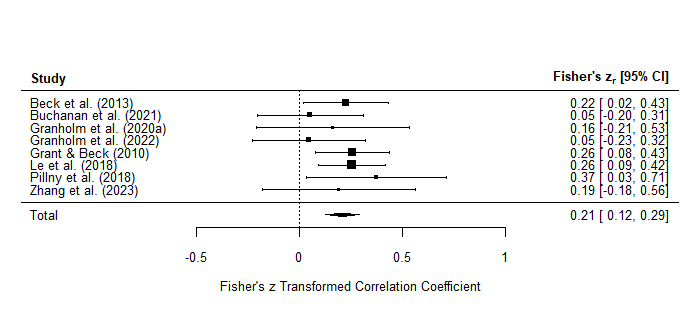
*

**Figure S4.** Forest plot of studies included in primary meta-analysis between asocial beliefs and overall negative symptoms. Effect sizes represented by *z*-transformed Pearson’s *r* correlations.

**Table S8.** Meta-analysis for relationship between asocial beliefs and total negative symptoms, as well as subset analyses for negative symptom subdomains.

|  | Number of Studies (*k*) | Number of Participants (*n*) | Effect Size (*r*) | 95% CI | *p* | SE | *Z* | *I^2^* | *τ^2^* |
| --- | --- | --- | --- | --- | --- | --- | --- | --- | --- |
| Total Negative Symptoms | 8 | 578 | 0.21 | 0.12 – 0.28 | <0.0001 | 0.04 | 4.9 | 0% | 0 |
| Diminished Motivation Subdomain | 5 | 215 | 0.15 | 0.02 – 0.28 | 0.03 | 0.08 | 2.0 | 0% | 0 |
| Diminished Expression Subdomain | 4 | 184 | 0.09 | -0.06 – 0.24 | 0.2 | 0.08 | 1.2 | 0% | 0 |

**Table S9.** Meta-regression analyses of moderators of relationship between overall negative symptoms and asocial beliefs.

|  | Number of Studies (*k*) | Number of Participants (*n*) | ß | SE | *p* |
| --- | --- | --- | --- | --- | --- |
| Age | 8 | 578 | -0.01 | 0.01 | 0.3 |
| Sex (% Male) | 8 | 578 | 0.001 | 0.004 | 0.8 |
| Years of Illness | 5 | 362 | -0.02 | 0.01 | 0.2 |
| Negative Symptom Severity | 8 | 578 | -0.03 | 0.05 | 0.5 |
| Study Quality | 8 | 578 | -0.04 | 0.05 | 0.5 |

**Table S10.** Subset analysis for each measure of asocial beliefs (minimum *k* = 3) included in the primary meta-analysis.

|  | Number of Studies (*k*) | Number of Participants (*n*) | Effect Size (*r*) | 95% CI | *p* | SE | *Z* | *I^2^* | *τ^2^* |
| --- | --- | --- | --- | --- | --- | --- | --- | --- | --- |
| ABS | 6 | 511 | 0.20 | 0.11 – 0.28 | <0.0001 | 0.05 | 4.4 | 0% | 0 |

Abbreviations: ABS: Asocial Beliefs Scale.

**Table S11.** Subset analysis for each negative symptom measure (minimum *k* = 3) included in the primary meta-analysis for asocial beliefs.

|  | Number of Studies (*k*) | Number of Participants (*n*) | Effect Size (*r*) | 95% CI | *p* | SE | *Z* | *I^2^* | *τ^2^* |
| --- | --- | --- | --- | --- | --- | --- | --- | --- | --- |
| SANS | 4 | 334 | 0.17 | 0.07 – 0.28 | 0.002 | 0.06 | 3.2 | 0% | 0 |

Abbreviations: SANS: Scale for the Assessment of Negative Symptoms.


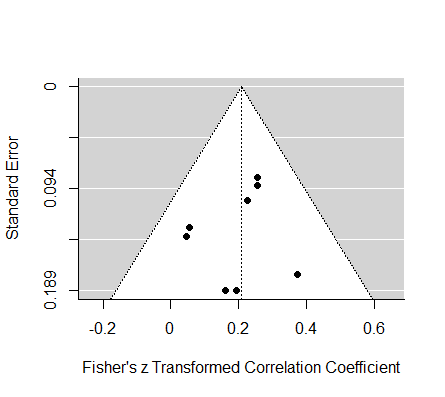


**Figure S5.** Funnel plot for primary meta-analysis of asocial beliefs.


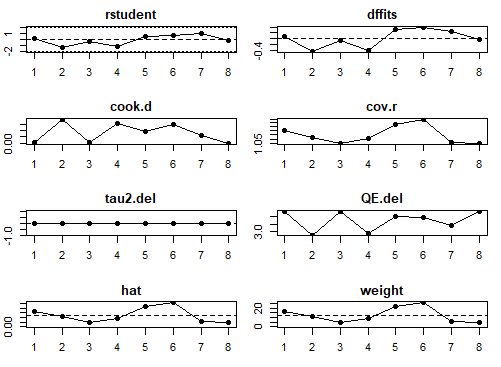


**Figure S6.** Influential outlier analyses for studies included in the primary meta-analysis for asocial beliefs (*k* = 8). Significant studies are illustrated by presence of red dots.

**Low Expectancies for Success**

**Table S12.** Summary of studies included in primary meta-analysis for low expectancies for success.

| Study | N | Country | Diagnosis | Status | Illness Duration – Years *M* (SD) | Age *M* (SD) | % Male | Negative Symptom Severity | Negative Symptom Measure | Expectancies for Success Measure |
| --- | --- | --- | --- | --- | --- | --- | --- | --- | --- | --- |
| Avery et al. (2009)^a^ | 50 | Australia | SSD | Inpatients | 10.1^b^ | 34.7 (12.8) | 60% | 4 | SANS | CES |
| Beaudette et al. (2020) | 43 | USA | SZ/SA | Outpatients |  | 44.9 (11.2) | 67.4% |  | PANSS | RSES |
| Best et al. (2019) | 70 | Canada | SSD+ | Outpatients | 10.8 (13.5)^c^ | 37.6 (16.3) | 78.6% | 2.5 | BPRS | GSES |
| Caqueo-Urízar et al. (2022) | 217 | Chile | SZ | Outpatients | 19.7 | 41.1 (16.34) | 57% | 1.5 | PANSS | RAS |
| Cardenas et al. (2013) | 97 | USA | SZ/SA | Outpatients |  | 50.9 (6.5) | 56.7% | 1.9 | PANSS | RSES |
| Cavelti et al. (2017) | 156 | Switzerland | SZ/SA | Outpatients | 17.9 (11.7) | 44.5 (11.7) | 66% |  | PANSS | RAS |
| Chang et al. (2015) | 351 | China | SSD | Outpatients | 3.1 (4.6) | 38.3 | 43.9% | 0.8 | PANSS | GSES |
| Cheng et al. (2023) | 26 | Canada | SSD |  | ≤ 2^d^ | 17 | 46% |  | PANSS | RAS |
| Chino et al. (2009)^e^ | 36 | Japan | SZ | Outpatients | 5.5 (3.9) | 28 (5) | 58.3% | 3.05 | PANSS | SECL |
| Choi et al. (2010) | 70 | USA | SZ/SA | Outpatients | 12.1 (7) | 38.5 (5.7) | 62% | 7 | BPRS | PCS |
| Chrostek et al. (2016) | 207 | Poland | SSD | Mixed (I) | 12.9 (11) | 38.3 (12.6) | 50.2% | 3 | BPRS | GSES |
| Clari et al. (2022) | 66 | Tanzania | SSD | Outpatients | 9.1 (8.1) | 33 (8.2) | 66.7% | 1 | PANSS | GSES |
| Cowan et al. (2023) | 29 | USA | SSD | Outpatients | 1.4 (1.3) | 22.4 (4) | 79.3% | 1.9 | PANSS | RSES |
| Fiszdon et al. (2016) | 64 | USA | SZ | Outpatients | 25.2 (11.4) | 47.8 | 56.3% | 1.1 | PANSS | PCS |
| Galliot et al. (2022) | 67 | France | SZ/SA | Outpatients | 19.7 (10.5) | 43.9 (1.3) | 77.6% | 3.4 | BPRS | RAS |
| García-Mieres et al. (2022) | 83 | USA | SSD | Outpatients |  | 49.7 (10.7) | 95% | 3.9 | PANSS | RAS |
| González-Domínguez et al. (2019) | 63 | Spain | SSD | Outpatients | 23.5 (11.2) | 48.1 (10.2) | 65.1% | 3.2 | CRD-PSS | RAS |
| Gruber et al. (2020) | 138 | Austria | SSD+ | Mixed | 15.1 (15) | 36.4 (11.3) | 53.6% | 3.3 | PANSS | ES |
| Haugen et al. (2021) | 66 | Norway | SSD |  | 5.2 (3.8) | 25.5 (6.6) | 60.6% | 2.6 | PANSS | GSES |
| Hayward et al. (2009) | 35 | England | SZ |  |  | 38 (11.8) | 60% | 2.9 | PANSS | ES |
| Herpertz et al. (2022) | 46 | Germany | SSD | Inpatients | 14.2 | 39.2 (13.8) | 65.3% | 3.4 | PANSS | GSES |
| Hill & Startup (2013) | 60 | Australia | SSD | Inpatients | 12.45^f^ | 34.4 | 73.3% | 7.7 | SANS | SEQ |
| Huddy et al. (2016) | 30 | England | SZ/SA | Mixed (O) |  | 39.4 (9.1) | 80% | 1.2 | PANSS | PE |
| Izydorczyk et al. (2019) | 201 | Poland | SZ |  | 10 | 29 | 52.7% |  | PANSS | RSA |
| Keefe et al. (2012) | 53 | USA | SZ | Outpatients |  | 37 (10.3) | 73.6% | 1.5 | PANSS | PCS |
| Kinoshita et al. (2023) | 49 | Japan | SZ | Outpatients | 19.5 | 48.5 | 77.6% | 3 | PANSS | RAS |
| Kukla et al. (2021) | 21 | USA | SZ/SA | Outpatients | 27 (20.8)^g^ | 51.1 (13.2) | 95.2% | 1.7 | PANSS | RAS |
| Kurtz et al. (2013) | 68 | USA | SZ/SA | Mixed (O) | 10.6 (9.8) | 31.4 (11.5) | 73.5% | 2.9 | PANSS | RSES |
| Laxmi et al. (2023) | 103 | India | SSD | Outpatients | 3.1 (4.1) | 26.9 (6.9) | 63.1% | 2.6 | NSA-16 | RAS |
| Lee et al. (2017) | 43 | Korea | SZ | Mixed (O) | 15.4 (8.2) | 42.9 (7.4) | 65.1% | 2.9 | PANSS | PCS |
| Lee et al. (2021) | 52 | Australia | SZ/SA | Outpatients | 14.3 (8.4) | 41.3 (9.7) | 67.3% | 2.2 | PANSS | RSES |
| Li et al. (2023) | 91 | Taiwan | SSD | Outpatients | 22 (9) | 47.4 (9.4) | 37.4% | 3.4 | BPRS | RAS |
| Lim et al. (2021) | 95 | Singapore | SZ/SA | Outpatients | 15.3 (8.9) | 40.9 (10.3) | 51.6% | 1.1 | PANSS | ES |
| Luther et al. (2015) | 118 | USA | SZ/SA | Outpatients |  | 47.7 (8.9) | 80% | 2.3 | PANSS | RAS |
| Lysaker et al. (2001)^h^ | 49 | USA | SZ/SA | Outpatients | 22^b^ | 44 | 96% |  | PANSS | AQ |
| Markiewicz & Dobrowolska (2021) | 44 | Poland | SZ | Outpatients | 11.4 (7.2) | 36.7 (7.9) | 100% | 1.8 | PANSS | GSES |
| Melau et al. (2015) | 316 | Denmark | SSD | Outpatients |  | 25.3 (4.2) | 49.7% | 2.8 | SANS | GSES |
| Murphy et al. (2023) | 16 | Canada | SSD+ | Outpatients | ≤ 2 | 23.5 (3.1) | 70.6% | 1.2 | BPRS | PCS |
| Norman et al. (2013) | 84 | Canada | SSD+ | Outpatients | 4.7 (3.7)^i^ | 28 (7.4) | 69% | 1.4 | SANS | RAS |
| Pratt et al. (2005) | 85 | USA | SZ/SA | Outpatients |  | 37.9 (9.1) | 62.4% |  | SANS | RSES |
| Priebe et al. (2015) | 179 | England | SSD+ | Outpatients | 12^d^ | 41.6 (10.1) | 69% | 2.5 | PANSS | GSES |
| Raffard et al. (2014) | 30 | France | SZ | Outpatients | 9.8 (8.9) | 28.9 (8.7) | 73.3% | 2.2 | PANSS | MSEQ |
| Rossi et al. (2017) | 921 | Italy | SZ | Outpatients | 16.2 | 40.2 (10.7) | 69.6% | 4.6 | BNSS | RSA |
| Santosh & Kundu (2023) | 150 | India | SZ | Outpatients | 3.1 (1.7) | 32 (8.5) | 69.3% | 3.5 | PANSS | CSE |
| Senormanci et al. (2021) | 39 | Turkey | SZ | Inpatient | 13.5 | 41.7 | 61.5% | 4.6 | SANS | RSA |
| Senormanci et al. (2022) | 139 | Turkey | SZ | Outpatient | 11.2 | 37 (10.3) | 52.5% | 2.3 | PANSS | RSA |
| Song et al. (2013) | 33 | Korea | SZ | Mixed | .8 (.8) | 21.4 (3.6) | 42% | 3.2 | SANS | SES |
| Strauss et al. (2015) | 46 | USA | SZ/SA | Outpatients |  | 41.7 (10.7) | 60.9% |  | BNSS | WMT-SE |
| Thonon et al. (2020) | 18 | Belgium | SZ/SA | Mixed (O) | 11.1 (6) | 36.3 (8.2) | 89% | 5.1 | BNSS | GSES |
| Vaskinn et al. (2015) | 51 | Norway | SZ/SA | Mixed (O) | 5.7 (5.8) | 27.9 (7.9) | 62.75% | 1.8 | PANSS | RSES^j^ |
| Vauth et al. (2007) | 172 | Germany | SZ | Outpatients | 15.6 (11.2) | 39.6 (11) | 60.5% |  | PANSS | GSES |
| Ventura et al. (2014) | 71 | USA | SSD | Outpatients | .5 (.5) | 21.7 (3.3) | 80% | 4 | SANS | RSES^k^ |
| Watanabe et al. (2022) | 98 | Taiwan | SZ |  | 15.7 (11.3) | 48.7 (12.8) | 50% | 3.8 | PANSS | RAS |
| Wciórka et al. (2015) | 110 | Poland | SSD+ | Mixed (O) | 13 (10.5) | 38.3 (11.4) | 39% | 2.9 | BPRS | GSES |
| Wright et al. (2021) | 103 | USA | SZ/SA | Outpatients | 29.1 (11) | 58.8 (7.2) | 54% | 5.0 | SANS | RSES^k^ |

Abbreviations: AQ: Attitude Questionnaire – Self-Efficacy; BNSS: Brief Negative Symptom Scale; BPRS: Brief Psychiatric Rating Scale; CES: Cognitive Expectancy Scale – Self-Efficacy; CSE: Cognitive Self-Efficacy Scale; CRD-PSS: Clinician-Rated Dimensions of Psychosis Symptom Severity; ES: Empowerment Scale – Self-Efficacy; GSES: General Self-Efficacy Scale; Mixed (I): Majority Inpatients; Mixed (O): Majority Outpatients; MSEQ: Memory Self-Efficacy Questionnaire; NSA-16: Negative Symptom Assessment ; PANSS: Positive and Negative Syndrome Scale; PCS: Perceived Competency Scale; PE: Performance Expectancy; RAS: Recovery Assessment Scale – Success and Goal Orientation; RSA: Resilience Scale for Adults – Personal Strength; RSES: Revised Self-Efficacy Scale; SA: Schizoaffective Disorder; SANS: Scale for the Assessment of Negative Symptoms; SECL: Self-Efficacy for Community Life Scale; SEQ: Self-Efficacy Questionnaire; SES: Self-Efficacy Scale (General); SSD: Schizophrenia-Spectrum Disorder; SSD+: Schizophrenia-Spectrum Disorder + Other Diagnoses (≤ 15%); SZ: Schizophrenia; WMT-ES: Word Memory Test – Success Expectancy.

^a^Partial correlation (controlling for depression) used for effect size.

^b^Calculated by subtracting mean age of first hospitalization from mean age.

^c^Derived from Best et al. (2020), represents duration (years) since diagnosis.

^d^Represents number of years of accessing services.

^e^Spearman’s correlation used for effect size.

^f^Calculated by subtracting mean age of first contact with mental health services from mean age.

^g^Represents number of years since first hospitalization,

^h^Value of nonsignificant correlation not reported; imputed with a correlation coefficient of zero.

^i^Represents number of years in treatment.

^j^Social self-efficacy subdomain only.

^k^Total score including self-efficacy for managing positive symptoms.

**Table S12.1.** Summary of studies included in subdomain analyses for low expectancies for success.

| Study | N | Diagnosis | Diminished Motivation Measure | Diminished Expression Measure | Expectancies for Success Measure |
| --- | --- | --- | --- | --- | --- |
| Avery et al. (2009) | 50 | SSD | SANS Diminished Motivation | SANS Diminished Expression | CES |
| Bentall et al. (2010)^a^ | 56 | SZ/SA | SANS Avolition |  | TMQ |
| Chang et al. (2017)^b^ | 321 | SSD | SANS Amotivation | SANS Diminished Expression | GSES |
| Chino et al. (2009)^c^ | 36 | SZ | PANSS Amotivation |  | SECL |
| Choi et al. (2012) | 40 | SZ/SA | Motivational Trait Questionnaire |  | PCS |
| Fisher et al. (2023) | 100 | SSD+ | MAP-SR |  | MSQ |
| Laxmi et al. (2023) | 103 | SSD | NSA-16 Diminished Motivation^d^ | NSA-16 Diminished Expression^e^ | RAS |
| Melau et al. (2015) | 307 | SSD | SANS Diminished Motivation | SANS Diminished Expression | GSES |
| Morgades-Bamba et al. (2019) | 167 | SZ | SQLS Motivation & Energy |  | GSES |
| Norman et al. (2013) | 84 | SSD+ | SANS Diminished Motivation | SANS Diminished Expression | RAS |
| Rossi et al. (2017) | 911 | SZ | BNSS-MAP | BNSS-EXP | RSA |
| Song et al. (2013) | 33 | SZ | SANS Diminished Motivation | SANS Diminished Expression | SES |
| Thonon et al. (2020) | 18 | SZ/SA | BNSS-MAP | BNSS-EXP | GSES |
| Ventura et al. (2014) | 71 | SSD | SANS Experiential | SANS Expressive | RSES |

Abbreviations: BNSS: Brief Negative Symptom Scale; CES: Cognitive Expectancy Scale – Self-Efficacy; GSES: General Self-Efficacy Scale; EXP: Expressivity subscale; MAP: Motivation and Pleasure subscale;MAP-SR: Motivation and Pleasure Scale – Self-Report; MSQ: Motivation State Questionnaire – Self-Efficacy; NSA-16: Negative Symptom Assessment; PCS: Perceived Competency Scale; RAS: Recovery Assessment Scale – Success and Goal Orientation; RSA: Resilience Scale for Adults – Personal Strength; RSES: Revised Self-Efficacy Scale; SA: Schizoaffective Disorder; SANS: Scale for the Assessment of Negative Symptoms; ; SECL: Self-Efficacy for Community Life Scale; SQLS: Schizophrenia Quality of Life Scale; SSD: Schizophrenia-Spectrum Disorder; SSD+: Schizophrenia-Spectrum Disorder + Other Diagnoses (≤ 15%); SZ: Schizophrenia; TMQ: Task Motivation Questionnaire.

^a^Effect size (*r*) was transformed from Cohen’s *d,* which was calculated using the reported means and standard deviations for avolition and non-avolition patients.

^b^Subset of Chang et al. (2015); only included in subdomain analysis.

^c^Spearman’s correlation used for effect size.

^d^Comprised of Motivation and Social Involvement subscales.

^e^Comprised of Communication, Affect, and Retardation subscales.

**
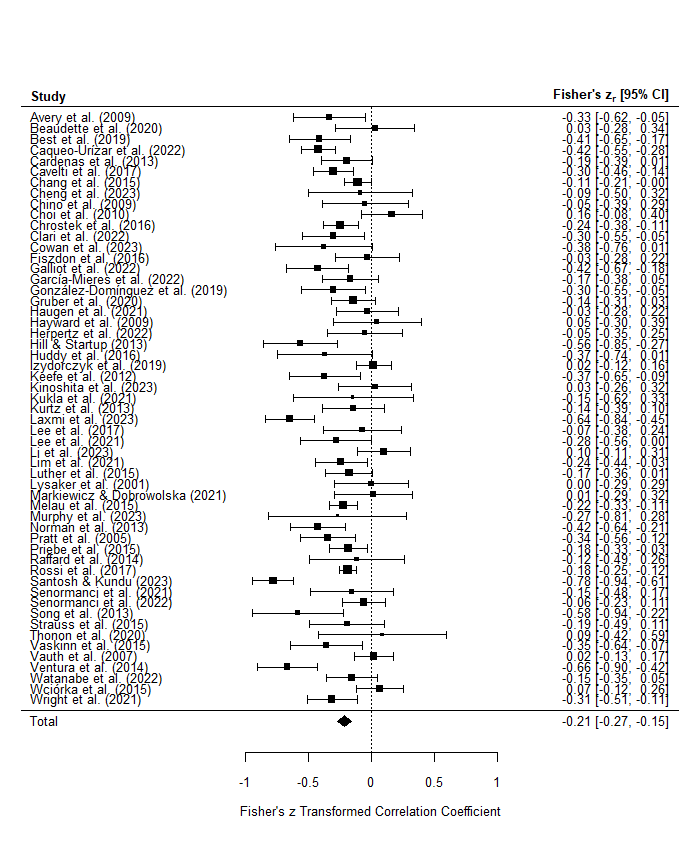
**

**Figure 7.** Forest plot of studies included in primary meta-analysis between low expectancies for success and overall negative symptoms. Effect sizes represented by *z*-transformed Pearson’s *r* correlations.

**Table S13.** Meta-analysis for relationship between low expectancies for success and total negative symptoms, as well as subset analyses for negative symptom subdomains.

|  | Number of Studies (*k*) | Number of Participants (*n*) | Effect Size (*r*) | 95% CI | *p* | SE | *Z* | *I^2^* | *τ^2^* |
| --- | --- | --- | --- | --- | --- | --- | --- | --- | --- |
| Total Negative Symptoms | 55 | 5664 | -0.21 | -0.15 – -0.26 | <0.0001 | 0.03 | -7.3 | 74.2% | 0.03 |
| Diminished Motivation Subdomain | 14 | 2297 | -0.33 | -0.22 – -0.33 | <0.0001 | 0.06 | -5.9 | 81.4% | 0.03 |
| Diminished Expression Subdomain | 9 | 1898 | -0.21 | -0.06 – -0.34 | 0.005 | 0.07 | -2.8 | 85.7% | 0.04 |

**Table S14.** Meta-regression analyses of moderators of relationship between overall negative symptoms and low expectancies for success.

|  | Number of Studies (*k*) | Number of Participants (*n*) | ß | SE | *p* |
| --- | --- | --- | --- | --- | --- |
| Age | 55 | 5664 | 0.007 | 0.003 | 0.03 |
| Sex (% Male) | 55 | 5664 | -0.002 | 0.002 | 0.2 |
| Years of Illness | 43 | 4732 | 0.01 | 0.005 | 0.02 |
| Negative Symptom Severity | 46 | 4825 | 0.0008 | 0.02 | 0.9 |
| Study Quality | 55 | 5664 | 0.02 | 0.03 | 0.4 |

**Table S15.** Subset analyses for each measure of low expectancies for success (minimum *k* = 3) included in primary meta-analysis.

|  | Number of Studies (*k*) | Number of Participants (*n*) | Effect Size (*r*) | 95% CI | *p* | SE | *Z* | *I^2^* | *τ^2^* |
| --- | --- | --- | --- | --- | --- | --- | --- | --- | --- |
| ES | 3 | 262 | -0.15 | -0.03 – -0.27 | 0.02 | 0.06 | -2.4 | 0.02% | 0 |
| GSES | 12 | 1632 | -0.13 | -0.05 – -0.21 | 0.001 | 0.04 | -3.2 | 54.9% | 0.01 |
| PCS | 5 | 244 | -0.09 | 0.11 – -0.28 | 0.4 | 0.10 | -0.9 | 53.9% | 0.03 |
| RAS | 13 | 1172 | -0.25 | -0.14 – -0.35 | <0.0001 | 0.06 | -4.3 | 72.3% | 0.03 |
| RSA | 4 | 1288 | -0.10 | 0.02 – -0.21 | 0.09 | 0.06 | -1.7 | 58% | 0.007 |
| RSES | 9 | 594 | -0.29 | -0.17 – -0.39 | <0.0001 | 0.06 | -4.8 | 51.6% | 0.02 |

Abbreviations: ES: Empowerment Scale – Self-Efficacy; GSES: General Self-Efficacy Scale; PCS: Perceived Competency Scale; RAS: Recovery Assessment Scale – Success and Goal Orientation; RSA: Resilience Scale for Adults – Personal Strength; RSES: Revised Self-Efficacy Scale.

**Table S16.** Subset analyses for each negative symptom measure (minimum *k* = 3) included in primary meta-analysis for low expectancies for success.

|  | Number of Studies (*k*) | Number of Participants (*n*) | Effect Size (*r*) | 95% CI | *p* | SE | *Z* | *I^2^* | *τ^2^* |
| --- | --- | --- | --- | --- | --- | --- | --- | --- | --- |
| BNSS | 3 | 975 | -0.18 | -0.12 – -0.24 | <0.0001 | 0.03 | -5.6 | 0% | 0 |
| BPRS | 7 | 631 | -0.13 | 0.06 – -0.31 | 0.2 | 0.10 | -1.4 | 80.1% | 0.05 |
| PANSS | 34 | 3074 | -0.17 | -0.10 – -0.23 | <0.0001 | 0.03 | -5.1 | 65.7% | 0.02 |
| SANS | 9 | 818 | -0.36 | -0.26 – -0.46 | <0.0001 | 0.06 | -6.7 | 52.1% | 0.01 |

Abbreviations: BNSS: Brief Negative Symptom Scale; BPRS: Brief Psychiatric Rating Scale; PANSS: Positive and Negative Syndrome Scale; SANS: Scale for the Assessment of Negative Symptoms.


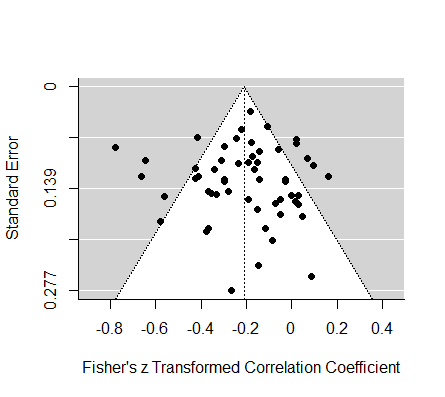
**Figure S8.** Funnel plot for primary meta-analysis of low expectancies for success.


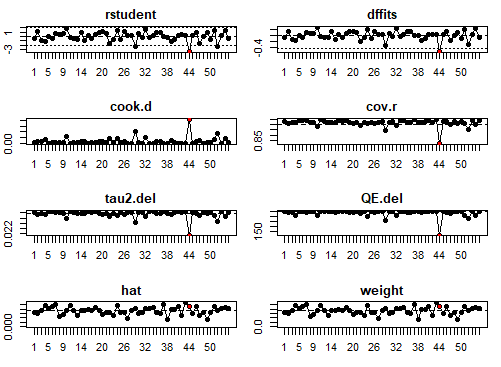


**Figure S9.** Influential outlier analyses for studies included in the primary meta-analysis of low expectancies for success (*k* = 55). Significant outlier study illustrated by red dot (Santosh & Kundu, 2023).

**Table S17.** Outlier study excluded: updated meta-analysis for relationship between low expectancies for success and total negative symptoms.

|  | Number of Studies (*k*) | Number of Participants (*n*) | Effect Size (*r*) | 95% CI | *p* | SE | *Z* | *I^2^* | *τ^2^* |
| --- | --- | --- | --- | --- | --- | --- | --- | --- | --- |
| Total Negative Symptoms | 54 | 5514 | -0.20 | -0.15 – -0.24 | <0.0001 | 0.03 | -7.5 | 67.3% | 0.02 |

Note: Subdomain analyses unaffected by excluded study.

**Table S18.** Outlier study excluded: updated meta-regression analyses.

|  | Number of Studies (*k*) | Number of Participants (*n*) | ß | SE | *p* |
| --- | --- | --- | --- | --- | --- |
| Age | 54 | 5514 | 0.006 | 0.003 | 0.05 |
| Sex (% Male) | 54 | 5514 | -0.002 | 0.002 | 0.2 |
| Years of Illness | 42 | 4582 | 0.009 | 0.005 | 0.07 |
| Negative Symptom Severity | 45 | 4675 | 0.005 | 0.02 | 0.8 |
| Study Quality | 54 | 5614 | -0.001 | 0.02 | 0.9 |

**Table S19.** Outlier study excluded: updated subset analysis for measures of negative symptoms.

|  | Number of Studies (*k*) | Number of Participants (*n*) | Effect Size (*r*) | 95% CI | *p* | SE | *Z* | *I^2^* | *τ^2^* |
| --- | --- | --- | --- | --- | --- | --- | --- | --- | --- |
| PANSS | 33 | 2924 | -0.15 | -0.10 – -0.20 | <0.0001 | 0.03 | -5.7 | 38.9% | 0.008 |

Abbreviations: PANSS: Positive and Negative Syndrome Scale.


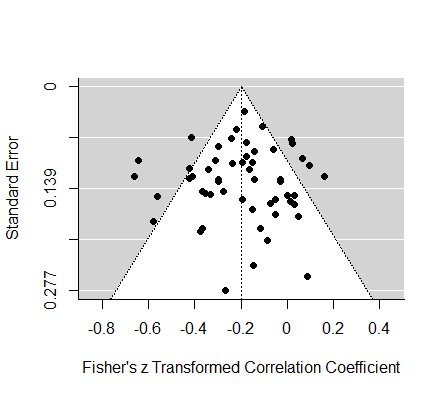


**Figure S10.** Outlier study excluded: updated funnel plot for primary meta-analysis of low expectancies for success.

**Low Expectancies for Pleasure**

**Table S20.** Summary of studies included in primary meta-analysis for low expectancies for pleasure.

| Study | N | Country | Diagnosis | Status | Illness Duration – Years *M* (SD) | Age *M* (SD) | % Male | Negative Symptom Severity | Negative Symptom Measure | Expectancies for Pleasure Measure |
| --- | --- | --- | --- | --- | --- | --- | --- | --- | --- | --- |
| Beck et al. (2013)^a^ | 93^b^ | USA | SZ | Outpatients | 17.9 | 39.5 | 75.5% | 2.8 | SANS | Low Expectations of Future Pleasure |
| Hartmann et al. (2015) | 29^c^ | Switzerland | SZ/SA | Mixed (I) |  | 30.4 (8.7) | 80.6% | 3.7 | BNSS^d^ | Anticipated Monetary Reward Pleasure |
| Hu et al. (2022) | 36 | China | SZ | Outpatients | 3.1 (4.1) | 23.1 (6.2) | 52.8% | 2.2 | SANS | BAPS |
| Strauss et al. (2015) | 46 | USA | SZ/SA | Outpatients |  | 41.7 (10.7) | 60.9% |  | BNSS | WMT-EE |
| Yang et al. (2018) | 45 | China | SZ | Mixed (I) | 16.5 (11.3) | 40.5 (11.9) | 62.2% | 2.1 | SANS | BAPS |

Abbreviations: BAPS: Beliefs about Pleasure Scale; BNSS: Brief Negative Symptom Scale; Mixed (I): Majority Inpatients; SA: Schizoaffective Disorder; SANS: Scale for the Assessment of Negative Symptoms; SZ: Schizophrenia; SSD: Schizophrenia-Spectrum Disorder; SSD+: Schizophrenia-Spectrum Disorder + Other Diagnoses (≤ 15%); WMT: Word Memory Test – Expected Enjoyment.

^a^Effect size (*r*) calculated using adjusted means and standard errors from comparing difference in low expectations between high and low deficit syndrome participants, while controlling for depression.

^b^N=94 for demographic information.

^c^N=31 for demographic information.

^d^Only reported the BNSS apathy subscale score; included in primary meta-analysis for increased power (see Table S21).

| Study | N | Diagnosis | Diminished Motivation Measure | Expectancies for Pleasure Measure |
| --- | --- | --- | --- | --- |
| Hartmann et al. (2015) | 29 | SZ/SA | BNSS Apathy subscale | Anticipated Monetary Reward Pleasure |
| Hu et al. (2022) | 36 | SZ | SANS Diminished Motivation | BAPS |
| Yang et al. (2018) | 45 | SZ | SANS Diminished Motivation | BAPS |

**Table S20.1.** Summary of studies included in subdomain analysis for low expectancies for pleasure.

Abbreviations: BAPS: Beliefs about Pleasure Scale; BNSS: Brief Negative Symptom Scale; SA: Schizoaffective Disorder; SANS: Scale for the Assessment of Negative Symptoms; SZ: Schizophrenia.

Note: *k* < 3 for diminished expression subdomain.

*
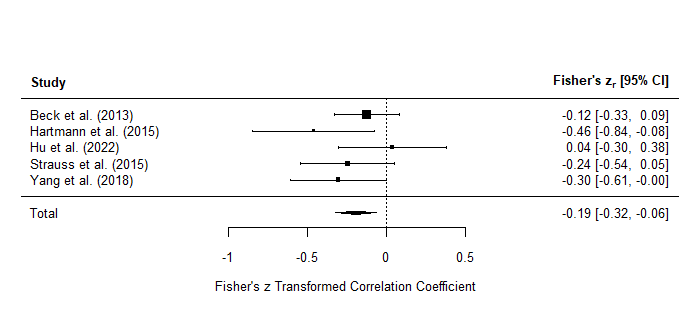
*

**Figure S11.** Forest plot of studies included in primary meta-analysis between low expectancies for pleasure and overall negative symptoms. Effect sizes represented by *z*-transformed Pearson’s *r* correlations.

**Table S21.** Meta-analysis for relationship between low expectancies for pleasure and total negative symptoms, as well as subset analysis for diminished motivation subdomain.

|  | Number of Studies (*k*) | Number of Participants (*n*) | Effect Size (*r*) | 95% CI | *p* | SE | *Z* | *I^2^* | *τ^2^* |
| --- | --- | --- | --- | --- | --- | --- | --- | --- | --- |
| Total Negative Symptoms | 5 | 249 | -0.19 | -0.06 – -0.31 | 0.003 | 0.07 | -2.9 | 0% | 0 |
| Total Negative Symptoms  Excluding Hartmann et al. | 4 | 220 | -0.16 | -0.02 – -0.29 | 0.02 | 0.07 | -2.3 | 0.02% | 0 |
| Diminished Motivation Subdomain | 3 | 110 | -0.21 | 0.10 – -0.48 | 0.2 | 0.16 | -1.3 | 61.2% | 0.05 |

**Table S22.** Meta-regression analyses of moderators of relationship between overall negative symptoms and low expectancies for pleasure.

|  | Number of Studies (*k*) | Number of Participants (*n*) | ß | SE | *p* |
| --- | --- | --- | --- | --- | --- |
| Age | 5 | 249 | -0.008 | 0.01 | 0.5 |
| Sex (% Male) | 5 | 249 | -0.007 | 0.009 | 0.4 |
| Years of Illness | 3 | 174 | -0.01 | 0.01 | 0.3 |
| Negative Symptom Severity | 4 | 203 | -0.2 | 0.2 | 0.3 |
| Study Quality | 5 | 249 | 0.02 | 0.06 | 0.7 |

**Table S23.** Subset analyses for each negative symptom measure (minimum *k* = 3) included in primary meta-analysis for low expectancies for pleasure.

|  | Number of Studies (*k*) | Number of Participants (*n*) | Effect Size (*r*) | 95% CI | *p* | SE | *Z* | *I^2^* | *τ^2^* |
| --- | --- | --- | --- | --- | --- | --- | --- | --- | --- |
| SANS | 3 | 174 | -0.13 | 0.02 – -0.28 | 0.08 | 0.08 | -1.7 | 0.01% | 0 |

Abbreviations: Scale for the Assessment of Negative Symptoms.

**
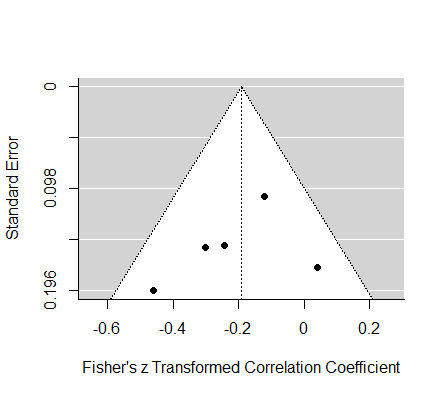
**

**Figure S12.** Funnel plot for primary meta-analysis of low expectancies for pleasure.


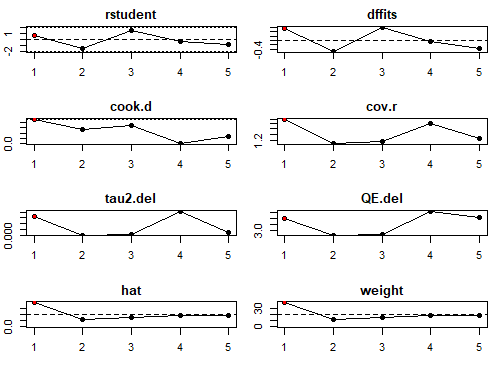


**Figure S13.** Influential outlier analyses for studies included in the primary meta-analysis of low expectancies for pleasure (*k* = 5). Significant outlier study illustrated by red dot (Beck, Grant, Huh, Perivoliotis, & Chang, 2013).

**Table S24.** Outlier study excluded: updated meta-analysis for relationship between low expectancies for pleasure and total negative symptoms.

|  | Number of Studies (*k*) | Number of Participants (*n*) | Effect Size (*r*) | 95% CI | *p* | SE | *Z* | *I^2^* | *τ^2^* |
| --- | --- | --- | --- | --- | --- | --- | --- | --- | --- |
| Total Negative Symptoms | 4 | 156 | -0.23 | -0.05 – -0.39 | 0.01 | 0.09 | -2.5 | 18.5% | 0.006 |

Note: Subdomain analyses unaffected by excluded study.

**Table S25.** Outlier study excluded: updated meta-regression analyses.

|  | Number of Studies (*k*) | Number of Participants (*n*) | ß | SE | *p* |
| --- | --- | --- | --- | --- | --- |
| Age | 4 | 156 | -0.01 | 0.01 | 0.3 |
| Sex (% Male) | 4 | 156 | -0.02 | 0.009 | 0.08 |
| Negative Symptom Severity | 3 | 110 | -0.2 | 0.2 | 0.4 |
| Study Quality | 4 | 156 | 0.1 | 0.08 | 0.2 |

Note: Duration of illness analysis unaffected by excluded study.

**
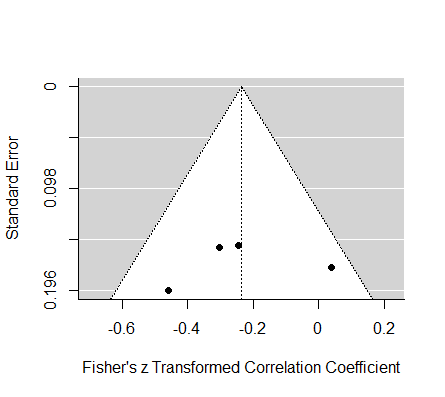
**

**Figure S14.** Outlier study excluded: updated funnel plot for primary meta-analysis of low expectancies for pleasure.

**Internalized Stigma**

**Table S26.** Summary of studies included in primary meta-analysis for internalized stigma.

| Study | N | Country | Diagnosis | Status | Age *M* (*SD*) | % Male | Illness Duration – Years *M* (*SD*) | Negative Symptom Severity | Negative Symptom Measure | Internalized Stigma Measure |
| --- | --- | --- | --- | --- | --- | --- | --- | --- | --- | --- |
| Acosta et al. (2020) | 133 | Spain | SZ | Outpatients | 46.7 (10.1) | 69.2% | 20.8 (11.2) | 2.5 | CGI-SCH | ISMI |
| Barlati et al. (2022) | 94 | Italy | SZ | Inpatients | 44.6 (11.4) | 77.7% | 19.1 (10.6) | 3.4 | PANSS | ISMI |
| Berry & Greenwood (2018) | 41^a^ | England | SSD+ | Outpatients | 26.1 (5.6) | 58.8% | 3 (3.6)^b^ | 1.6 | PANSS | ISMI |
| Campellone et al. (2014) | 50^a^ | USA | SZ/SA | Outpatients | 45.9 (10.6) | 52.9% | 21.9 | 3.9 | CAINS | ISMI |
| Căpățînă & Micluția (2018) | 106 | Romania | SZ | Outpatients | 41 (10.2) | 17% | 12.7 (9.6) | 4.7 | NSA-16 | ISMI |
| Caqueo-Urízar et al. (2019) | 253 | Chile, Peru, Bolivia | SZ | Outpatients | 35.6 (12.5) | 66.4% | 14.6 (11.8) | 2.8 | PANSS | ISMI-12 |
| Caqueo-Urízar et al. (2022) | 217 | Chile | SZ | Mixed | 41.1 (16.3) | 57% | 19.7 | 1.5 | PANSS | ISMI |
| Chan et al. (2019)^c^ | 64^d^ | China | SSD | Outpatients | 29.8 (10.1) | 59.4% | 4.4 (3.6) | 1.0 | PANSS | ISMI^e^ |
| Chen et al (2016) | 44 | China | SSD | Outpatients | 25.8 (9.7) | 52.3% |  | 1.1 | PANSS | SSS-S |
| Chrostek et al. (2016) | 207 | Poland | SSD | Mixed (I) | 38.3 (12.6) | 50.2% | 12.9 (11) | 3.0 | BPRS | ISMI |
| Chu et al. (2023) | 101 | China | SSD | Outpatient | 40.5 (7.8) | 42.6% | 5.2 | 1.5 | BNSS | SSS-S |
| Clari et al. (2022) | 66 | Tanzania | SSD | Outpatient | 33 (8.2) | 66.7% | 9.1 (8.1) | 1.0 | PANSS | ISMI |
| Cloutier et al. (2023) | 21 | Canada | SSD | Outpatient | 26.6 (5.1) | 100% |  | 3.7 | BPRS | ISMI |
| Degnan et al. (2022) | 46^f^ | UK | SSD | Mixed | 42.4 (13.0) | 70.6% | 17.3 (13.6) | 2.3 | PANSS | ISMI |
| DeLuca et al. (2021) | 13 | USA | SSD | Outpatients | 16.9 (2.5) | 56% |  | 3.7 | SIPS | ISMI |
| DeTore et al. (2022) | 40 | USA | SSD | Outpatients | 22.4 (3.0) | 80% | 3.1 | 1.9 | BPRS | ISMI |
| Fekih-Romdhane et al. (2023) | 202 | Lebanon | SZ/SA | Inpatients | 56.7 (11.6) | 62.4% | 31.6 (12.3) | 3.7 | SNS | ISMI |
| Feldhaus et al. (2018) | 81 | Germany | SZ/SA | Mixed (I) | 38.4 (13) | 54.3% | 11.8 (10.1) | 2.0 | PANSS | ISMI |
| Firmin et al. (2019) | 110^g^ | USA | SSD | Outpatients | 41.9 | 89.2% |  | 2.5 | PANSS | ISMI |
| González-Domínguez et al. (2019) | 63 | Spain | SSD | Outpatients | 48.1 (10.2) | 65.1% | 23.5 (11.2) | 3.2 | CRD-PSS | ISMI |
| Grover et al. (2017) | 707 | India | SZ |  | 33.9 (10.7) | 57.3% | 8.4 (7) | 0.7 | PANSS | ISMI |
| Grover et al. (2018) | 136 | India | SZ | Mixed | 33.4 (10) | 50.7% | 12 (7.2) | 2.1 | PANSS | ISMI |
| Gruber et al. (2020) | 130^h^ | Austria | SSD+ | Mixed | 36.4 (11.3) | 53.6% | 15.1 (15) | 3.2 | PANSS | ISMI |
| Hill & Startup (2013) | 60 | Australia | SSD | Inpatients | 34.4 | 73.3% | 12.5^i^ | 7.7 | SANS | ISMI |
| Hofer et al. (2016) | 110 | Austria & Japan | SZ | Outpatients | 45.2 | 42.9% | 17.3 (10.6) | 2.1 | PANSS | ISMI |
| Hofer et al. (2019)^j^ | 54 | Austria | SZ | Outpatients | 43.9 (10.5) | 50% | 14.8 (10.4) | 2.1 | PANSS | ISMI |
| Horsselenberg et al. (2016) | 102 | Netherlands | SSD | Outpatients | 39.1 (11.3) | 75.5% | 13.3 (10.3) | 1.7 | PANSS | ISMI |
| Huang et al. (2023) | 86 | Taiwan | SZ | Inpatients | 48.7 | 47.7% | 22.1 | 0.8 | PANSS | ISMI |
| İpçi et al. (2020) | 120 | Turkey | SZ/SA | Outpatients | 36.6 (8.4) | 76.7% | 13.6 (7.8) | 1.7 | PANSS | ISMI |
| Jian et al. (2022) | 300 | Taiwan | SZ/SA | Outpatients | 45.9 (11.7) | 46.3% | 18.9 (10.1) | 4.3 | PANSS | SSS-S |
| Karidi et al. (2014)^c^ | 100 | Greece | SZ | Outpatients | 39.7 (9.4) |  |  |  | PANSS | SIMI |
| Karidi et al. (2015) | 60 | Greece | SZ | Outpatients | 40 (9.8) | 67% | 22 (6) | 2.8 | PANSS | SIMI |
| Khalaf et al. (2023) | 50 | Egypt | SZ | Outpatients |  |  |  | 1.5 | PANSS | ISMI |
| Koçak et al. (2022) | 88 | Turkey | SZ | Outpatients | 35.1 (9.6) | 58% | 11.4 | 1.1 | PANSS | ISMI |
| Konsztowicz et al. (2021) | 40^k^ | Canada | SSD | Mixed (O) | 40.3 | 48.6% | 16.7 | 2.5 | SANS | ISMI |
| Krzyzanowski et al. (2021) | 29 | Canada | SZ/SA | Outpatients | 41.97 (8.6) | 55.2% | 16.9 (8.8) | 3.0 | SANS | ISMI-9 |
| Laxmi et al. (2023) | 103 | India | SSD | Outpatients | 26.9 (6.9) | 63.1% | 3.1 (1.4) | 2.6 | NSA-16 | ISMI |
| Li et al. (2017) | 384 | China | SZ | Outpatients | 40 (7.7) | 51.3% | 14.5 (8) | 2.4 | PANSS | ISMI |
| Lien et al. (2018) | 170 | Taiwan | SZ/SA | Outpatients | 44.4 (10.1) | 55% | 17.9 (10) | 3.0 | PANSS | ISMI^e^ |
| Lim et al. (2021) | 95 | Singapore | SZ/SA | Outpatients | 40.9 (10.3) | 51.6% | 15.3 (8.9) | 1.1 | PANSS | ISMI |
| Lo et al. (2022) | 86 | Switzerland | SSD | Inpatients | 40.6 (12.3) | 57% |  | 2.8 | BPRS | SSMIS-SF |
| Luciano et al. (2021) | 120 | Italy | SSD | Outpatients | 42.2 (11.2) | 55.8% | 16.8 (10.4) | 2.7 | BPRS | ISMI |
| Lv et al. (2013) | 92^l^ | China | SZ | Mixed | 26.3 (7.8) | 61.1% | 4.5 (3.9) | 3.1 | SANS | ISMI^e^ |
| Lysaker et al. (2007a) | 75 | USA | SZ/SA | Outpatients | 48.3 (7.0) | 85.3% | 23^m^ | 2.0 | PANSS | ISMI^e^ |
| Ma et al. (2023) | 95 | China | SZ | Inpatients | 37.1 | 49.5% |  |  | PANSS | ISMI |
| MacDougall et al. (2015) | 102 | Canada | SSD+ | Outpatients | 27 (7.4) | 70.6% | 4 (3.8)^n^ | 1.4 | SANS | SSMIS |
| Margetić et al. (2010) | 120 | Croatia | SZ | Outpatients | 33.9 (10.5) | 58% | 7.9 (7.3) | 2.6 | PANSS | ISMI |
| Murphy et al. (2023) | 16^o^ | Canada | SSD+ | Outpatients | 23.5 (3.1) | 70.6% | ≤ 2 | 1.2 | BPRS | PBIQ |
| Nabors et al. (2014) | 62 | USA | SZ/SA | Outpatients | 50.9 (10.6) | 95% | 21.8^m^ | 2.4 | PANSS | ISMI |
| Ng et al. (2024) | 179 | China | SSD | Outpatients | 31.7 (10.7) | 41.3% | 2.5 (1.3) | 1.6 | SANS | SSS-S |
| O’Connor et al. (2018) | 177 | USA | SZ/SA | Outpatients | 45.4 | 61% |  | 2.1 | PANSS | ISMI |
| Ordóñez-Camblor et al. (2021) | 114 | Spain | SSD+ | Outpatients | 35.5 (9.3) | 71.9% | 9.7 (7.7) | 6.5 | CAPE | ISMI |
| Park et al. (2013) | 49 | USA | SZ/SA | Outpatients | 49.6 (7.2) | 71.4% |  |  | SANS | ISMI^e^ |
| Pérez-Aguado et al. (2024) | 60 | Spain | SSD | Outpatients | 39.1 (9.5) | 80% |  | 4.4 | PANSS | ISMI |
| Pishdadian et al. (2023) | 57^p^ | Canada | SSD+ | Outpatients | 35.7 (11.8) | 62.7% | 10.5 | 3.0 | SANS | ISMI-10 |
| Pos et al. (2019) | 73^q^ | Netherlands | SSD | Mixed | 25.4 | 80.8% | < 4 | 3.7 | BNSS | ISMI |
| Prouteau et al. (2017) | 60 | France | SSD | Outpatients | 40.3 (10.2) | 83% | 14.2 (7.7) | 4.9 | PANSS | ISMI |
| Reneses et al. (2020) | 89 | Spain | SZ | Mixed (O) | 43.8 (11.4) | 75.3% | 20 (11.7) | 0.4 | PANSS | SSQ |
| Rossi et al. (2017) | 910^r^ | Italy | SZ | Outpatients | 40.2 (10.7) | 69.6% | 16.2^s^ | 4.6 | BNSS | ISMI |
| Schrank et al. (2014) | 284 | Austria | SSD | Mixed | 39.9 (12.6) | 58.1% | 15.2 (12.8) | 0.95 | PANSS | ISMI |
| Schwarzbold et al. (2021) | 98 | USA | SSD+ | Outpatients | 47.6 (13.2) | 80.6% | 21.2 (12.5) | 4.8 | CAINS | ISMI |
| Sen et al. (2020) | 30 | India | SSD | Outpatients | 27.4 (6.9) | 70% | 0.75^t^ | 1.6 | PANSS | ISMI |
| Shaheen & Amin (2016) | 50 | Pakistan | SZ/SA |  | 38 (7.3) | 68% | 7 (4.3) | 3.3 | PANSS | ISMI |
| Shin et al. (2016) | 70 | Korea | SZ | Outpatients | 35.8 (10.4) | 47.1% | 7.1 (2.8) |  | MS | ISMI |
| Singh et al. (2016) | 100 | India | SZ | Outpatients | 36.8 (12.1) | 54% | 10.2 (8.1) |  | PANSS | ISMI |
| Singla et al. (2020) | 100 | India | SZ | Outpatients | 37.1 (12.1) | 65% | 10.3 (7.6) | 0.4 | PANSS | ISMI |
| Staring et al. (2013) | 21 | Netherlands | SSD | Outpatients | 40.6 | 66.7% | 13 | 3.1 | PANSS | ISMI |
| Styla & Switaj (2024) | 86 | Poland | SZ | Mixed | 42.8 (11.4) | 72.1% | 19.4 (11.3) | 2.9 | BPRS | ISMI |
| Suman et al. (2023) | 160 | India | SZ | Outpatients | 35 (9.1) | 59.4% | 12.8 (7.6) | 0.8 | PANSS | ISMI |
| Swanson et al. (2022) | 15 | Scotland | SSD | Mixed (I) | 42.6 | 87% |  | 3.7 | BNSS | PBIQ |
| Świtaj et al. (2014) | 110 | Poland | SSD+ | Mixed | 38.4 (11.4) | 39.1% | 12.4 (10.5) | 2.9 | BPRS | ISMI |
| Tao et al. (2022) | 142 | China | SSD+ |  | 35.6 (6.9) | 45.8% |  | 3.1 | SANS | SSS-S |
| Tu et al. (2023) | 86 | Taiwan | SZ | Mixed | 46.6 (9.0) | 47.7% | 23.2^u^ |  | PANSS | ISMI^e^ |
| Villagonzalo et al. (2019) | 134^v^ | Australia | SSD | Outpatients | 40.4 (10.7) | 51.1% | 18.8^s^ | 0.9 | PANSS | ISMI |
| Vrbova et al. (2018) | 57 | Czechia | SSD | Outpatients | 35.8 (9.9) | 45.6% | 7.3 | 2.1 | PANSS | ISMI |
| White et al. (2007) | 100 | Ireland | SZ | Mixed (O) | 58.8 (7.2) | 78% | 29.1 (11) | 3.5 | SANS | PBIQ |
| White et al. (2023) | 187^w^ | UK | SSD | Mixed (O) | 37.5 (12.8) | 36.3% |  | 5.9 | CAPE | ISMI-10 |
| Yanos et al. (2008) | 100^x^ | USA | SZ/SA | Outpatients | 46.2 (9.6) | 85.3% |  |  | PANSS | ISMI |
| Yanos et al. (2012) | 30 | USA | SZ/SA | Outpatients | 46.9 | 77% | 22.7^y^ | 2.1 | PANSS | ISMI |
| Yildiz et al. (2019) | 162 | Turkey | SZ/SA | Outpatients | 35.6 (9.4) | 76.5% | 14.1 (8.6) | 2.3 | PANSS | SSIP |
| Zhang et al. (2019) | 232 | China | SZ |  |  | 59.1% |  |  | PANSS | ISMI^e^ |

Abbreviations: BNSS: Brief Negative Symptom Scale; BPRS: Brief Psychiatric Rating Scale; CAINS: Clinical Assessment Interview for Negative Symptoms; CAPE: Community Assessment of Psychic Experiences; CGI-SCH: Clinical Global Impression – Schizophrenia; CRD-PSS: Clinician-Rated Dimensions of Psychosis Symptom Severity; ISMI: Internalized Stigma of Mental Illness Scale; Mixed (I): Majority Inpatients; Mixed (O): Majority Outpatients; MS: Manchester Scale; NSA-16: Negative Symptom Assessment; PANSS: Positive and Negative Syndrome Scale; Personal Beliefs about Illness Questionnaire – Entrapment/Expectations; SA: Schizoaffective Disorder; SANS: Scale for the Assessment of Negative Symptoms; SIPS: Structured Interview for Psychosis-Risk Syndromes; SNS: Self-Evaluation of Negative Symptoms; SSD: Schizophrenia-Spectrum Disorder; SSD+: Schizophrenia-Spectrum Disorder + Other Diagnoses (≤ 15%); SSIP: Self-Stigma Inventory for Patients with Schizophrenia; SSQ: Self-Stigma Questionnaire; SSS-S: Self-Stigma Scale – Short; SSMIS: Self-Stigma of Mental Illness Scale; SSMIS-SF: Self-Stigma of Mental Illness Scale – Short Form; SIMI: Stigma Inventory of Mental Illness; SZ: Schizophrenia.

^a^N=51 for demographic information.

^b^Represents years of mental health service use.

^c^Spearman’s correlation used for effect size.

^d^N=16 for demographic information.

^e^Excluding Stigma Resistance subscale.

^f^N=50 for demographic information, except negative symptoms (N=47)

^g^N=111 for demographic information.

^h^N=138 for demographic information.

^i^Calculated by subtracting mean age of first contact with mental health services from mean age.

^j^Sample consists of 19 participants who were also included in Hofer et al. (2016), but with 18 months in between participation.

^k^N=35 for demographic information.

^l^N=95 for demographic information.

^m^Calculated by subtracting mean age of first psychiatric hospitalization from mean age.

^n^Represents time in treatment.

^o^N=17 for demographic information.

^p^N=59 for demographic information, except duration of illness (N=54)

^q^N=99 for demographic information.

^r^N=921 for demographic information.

^s^Calculated by subtracting mean age at onset of psychotic symptoms from mean age.

^t^Calculated by adding mean duration of untreated psychosis and mean duration of treatment.

^u^N=85 for duration of illness.

^v^N=135 for demographic information.

^w^N=190 for demographic information.

^x^N=102 for demographic information.

^y^Calculated by subtracting mean age of first psychiatric hospitalization from mean age, N=39 (including N=9 BP/MDD participants).

**Table S26.1.** Summary of studies included in subdomain analyses for internalized stigma.

| Study | N | Diagnosis | Diminished Motivation Measure | Diminished Expression Measure | Internalized Stigma Measure |
| --- | --- | --- | --- | --- | --- |
| Campellone et al. (2014) | 50 | SZ/SA | CAINS-MAP | CAINS-EXP | ISMI |
| Căpățînă & Micluția (2018) | 106 | SZ | NSA-16 Experiential | NSA-16 Expressive | ISMI |
| Chu et al. (2023) | 101 | SSD | BNSS-MAP | BNSS-EXP | SSS-S |
| DeTore et al. (2022) | 38 | SSD | QLS Intrinsic Motivation |  | ISMI |
| Fekih-Romdhane et al. (2023) | 202 | SZ/SA | SNS Diminished Motivation | SNS Diminished Expression | ISMI |
| Firmin et al. (2019) | 110 | SSD | QLS Intrapsychic Foundations |  | ISMI |
| Konsztowicz et al. (2020) | 40 | SSD | SANS Diminished Motivation | SANS Diminished Expression | ISMI |
| Krzyzanowski et al. (2021) | 29 | SZ/SA | SANS Diminished Motivation | SANS Diminished Expression | ISMI |
| Laxmi et al. (2023) | 103 | SSD | NSA-16 Diminished Motivation^d^ | NSA-16 Diminished Expression^e^ | ISMI |
| Li et al. (2017) | 384 | SZ | SQLS Motivation & Energy |  | ISMI |
| Lysaker et al. (2007b)^a^ | 36 | SZ/SA | QLS Intrapsychic Foundations |  | ISMI^b^ |
| MacDougall et al. (2015) | 102 | SSD+ | SANS Diminished Motivation | SANS Diminished Expression | SSMIS |
| Morgades-Bamba et al. (2019) | 167 | SZ | SQLS Motivation & Energy |  | ISMI^c^ |
| Ng et al. (2024) | 179 | SSD | SANS Diminished Motivation | SANS Diminished Expression | SSS-S |
| Park et al. (2013) | 49 | SZ/SA | SANS Diminished Motivation | SANS Diminished Expression | ISMI^b^ |
| Pishdadian et al. (2023) | 57 | SSD+ | SANS Diminished Motivation | SANS Diminished Expression | ISMI-10 |
| Pos et al. (2019) | 73 | SSD | BNSS-MAP | BNSS Blunted Affect | ISMI |
| Rossi et al. (2017) | 910 | SZ | BNSS-MAP | BNSS-EXP | ISMI |
| Schwarzbold et al. (2021) | 98 | SSD+ | CAINS-MAP | CAINS-EXP | ISMI |
| Suman et al. (2023) | 160 | SZ | SQLS Motivation & Energy |  | ISMI |
| Swanson et al. (2021) | 15 | SSD | BNSS-MAP | BNSS-EXP | PBIQ |
| Tao et al. (2022) | 142 | SSD+ | SANS Diminished Motivation | SANS Diminished Expression | SSS-S |
| Yanos et al. (2008) | 102 | SZ/SA | PANSS Social Avoidance |  | ISMI |

Abbreviations: BNSS: Brief Negative Symptom Scale; CAINS: Clinical Assessment Interview for Negative Symptoms; EXP: Expressivity subscale; ISMI: Internalized Stigma of Mental Illness Scale; MAP: Motivation and Pleasure subscale; NSA-16: Negative Symptom Assessment; PANSS: Positive and Negative Syndrome Scale; Personal Beliefs about Illness Questionnaire – Entrapment/Expectations; QLS: Quality of Life Scale; SA: Schizoaffective Disorder; SANS: Scale for the Assessment of Negative Symptoms; SNS: Self-Evaluation of Negative Symptoms; SSD: Schizophrenia-Spectrum Disorder; SSD+: Schizophrenia-Spectrum Disorder + Other Diagnoses (≤ 15%); SSMIS: Self-Stigma of Mental Illness Scale; SSS-S: Self-Stigma Scale – Short; SQLS: Schizophrenia Quality of Life Scale; SZ: Schizophrenia.

^a^Unable to confirm if this sample overlaps with Lysaker, Roe, & Yanos (2007); therefore, only using this study for subdomain analysis.

^b^Excluding Stigma Resistance subscale.

^c^Composite of Alienation & Stereotype Endorsement subscales only.

^d^Comprised of Motivation and Social Involvement subscales.

^e^Comprised of Communication, Affect, and Retardation subscales.

*
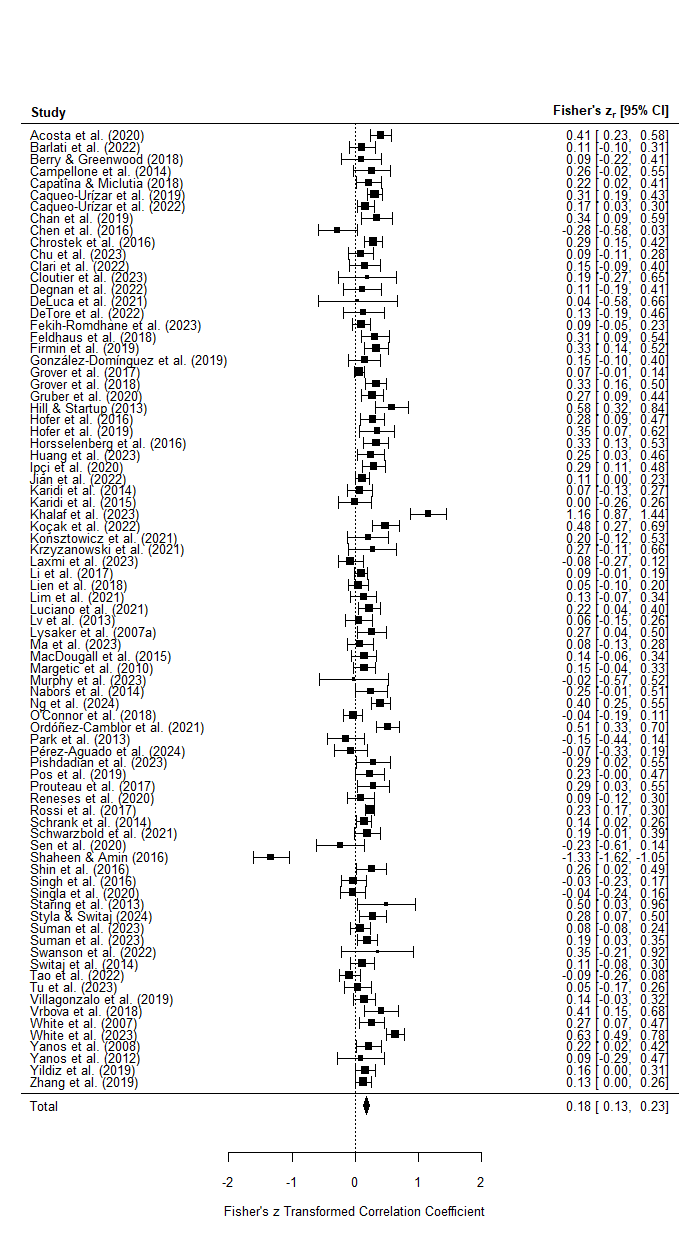
*

**Figure S15.** Forest plot of studies included in primary meta-analysis between internalized stigma and overall negative symptoms. Effect sizes represented by *z*-transformed Pearson’s *r* correlations.

**Table S27.** Meta-analysis for relationship between internalized stigma and total negative symptoms, as well as subgroup analyses for negative symptom subdomains.

|  | Number of Studies (*k*) | Number of Participants (*n*) | Effect Size (*r*) | 95% CI | *p* | SE | *Z* | *I^2^* | *τ^2^* |
| --- | --- | --- | --- | --- | --- | --- | --- | --- | --- |
| Total Negative Symptoms | 81 | 9766 | 0.17 | 0.12 – 0.22 | <0.0001 | 0.03 | 6.5 | 83.9% | 0.05 |
| Diminished Motivation Subdomain | 23 | 3255 | 0.21 | 0.15 – 0.28 | <0.0001 | 0.04 | 6.1 | 68.9% | 0.02 |
| Diminished Expression Subdomain | 16 | 2256 | 0.08 | -0.01 – 0.16 | 0.07 | 0.04 | 1.8 | 66.6% | 0.02 |

**Table S28.** Meta-regression analyses of moderators of relationship between overall negative symptoms and internalized stigma.

|  | Number of Studies (*k*) | Number of Participants (*n*) | ß | SE | *p* |
| --- | --- | --- | --- | --- | --- |
| Age | 79 | 9484 | 0.002 | 0.004 | 0.6 |
| Sex (% Male) | 79 | 9616 | -0.002 | 0.002 | 0.9 |
| Years of Illness | 63 | 8196 | 0.004 | 0.004 | 0.4 |
| Negative Symptom Severity | 74 | 9020 | 0.03 | 0.02 | 0.2 |
| Study Quality | 81 | 9766 | 0.06 | 0.03 | 0.08 |

**Table S29.** Subset analyses for each measure of internalized stigma (minimum *k* = 3) included in primary meta-analysis.

|  | Number of Studies (*k*) | Number of Participants (*n*) | Effect Size (*r*) | 95% CI | *p* | SE | *Z* | *I^2^* | *τ^2^* |
| --- | --- | --- | --- | --- | --- | --- | --- | --- | --- |
| ISMI - All Versions | 67 | 8270 | 0.19 | 0.13 – 0.25 | <0.0001 | 0.03 | 6.1 | 85.8% | 0.05 |
| ISMI - Full Versions Only | 56 | 6976 | 0.19 | 0.12 – 0.25 | <0.0001 | 0.04 | 5.3 | 87.1% | 0.06 |
| ISMI - Excluding Stigma Resistance | 7 | 768 | 0.11 | 0.03 – 0.18 | 0.007 | 0.04 | 2.7 | 13.3% | 0.002 |
| PBIQ | 3 | 131 | 0.24 | 0.07 – 0.40 | 0.007 | 0.09 | 2.7 | 0% | 0 |
| SSS-S | 5 | 766 | 0.07 | -0.14 – 0.27 | 0.5 | 0.11 | 0.6 | 87% | 0.05 |

Abbreviations: ISMI: Internalized Stigma of Mental Illness Scale; Personal Beliefs about Illness Questionnaire – Entrapment/Expectations; SSS-S: Self-Stigma Scale – Short.

**Table S30.** Subset analyses for each negative symptom measure (minimum *k* = 3) included in primary meta-analysis for internalized stigma.

|  | Number of Studies (*k*) | Number of Participants (*n*) | Effect Size (*r*) | 95% CI | *p* | SE | *Z* | *I^2^* | *τ^2^* |
| --- | --- | --- | --- | --- | --- | --- | --- | --- | --- |
| BNSS | 4 | 1099 | 0.22 | 0.16 – 0.27 | <0.0001 | 0.03 | 7.3 | 0% | 0 |
| BPRS | 8 | 686 | 0.19 | 0.10 – 0.27 | <0.0001 | 0.04 | 4.2 | 17.3% | 0.003 |
| PANSS | 48 | 5992 | 0.15 | 0.07 – 0.23 | 0.0002 | 0.04 | 3.8 | 88.5% | 0.06 |
| SANS | 10 | 850 | 0.19 | 0.06 – 0.32 | 0.006 | 0.07 | 2.8 | 73.2% | 0.03 |

Abbreviations: BNSS: Brief Negative Symptom Scale; BPRS: Brief Psychiatric Rating Scale; PANSS: Positive and Negative Syndrome Scale; SANS: Scale for the Assessment of Negative Symptoms.


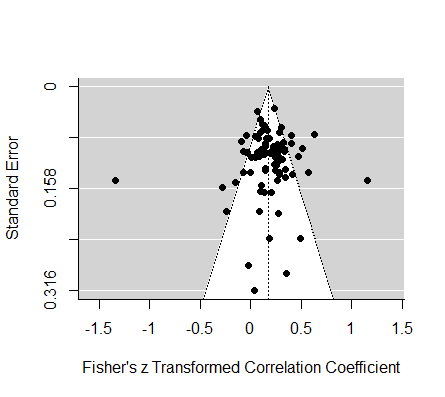


**Figure S16.** Funnel plot for primary meta-analysis of internalized stigma.


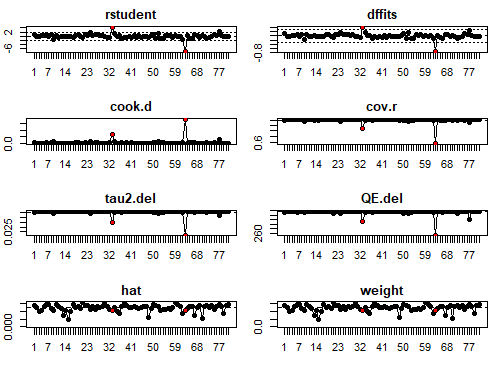


**Figure S17.** Influential outlier analyses for studies included in the primary meta-analysis of internalized stigma (*k* = 81). Significant outlier studies are illustrated by red dots (Khalaf, Fathy, Ebrahim, & Samie, 2023; Shaheen & Amin, 2016).

**Table S31.** Outlier studies excluded: updated meta-analysis for relationship between internalized stigma and total negative symptoms.

|  | Number of Studies (*k*) | Number of Participants (*n*) | Effect Size (*r*) | 95% CI | *p* | SE | *Z* | *I^2^* | *τ^2^* |
| --- | --- | --- | --- | --- | --- | --- | --- | --- | --- |
| Total Negative Symptoms | 79 | 9666 | 0.18 | 0.14 – 0.22 | <0.0001 | 0.02 | 9.8 | 64.0% | 0.02 |

Note: Subdomain analyses unaffected by excluded studies.

**Table S32.** Outlier studies excluded: updated meta-regression analyses.

|  | Number of Studies (*k*) | Number of Participants (*n*) | ß | SE | *p* |
| --- | --- | --- | --- | --- | --- |
| Age | 78 | 9434 | 0.001 | 0.003 | 0.7 |
| Sex (% Male) | 78 | 9566 | 0.0003 | 0.001 | 0.8 |
| Years of Illness | 62 | 8146 | -0.0003 | 0.003 | 0.9 |
| Negative Symptom Severity | 72 | 8920 | 0.04 | 0.01 | 0.004 |
| Study Quality | 81 | 9766 | 0.06 | 0.03 | 0.08 |

**Table S33.** Outlier studies excluded: subset analyses for measures of internalized stigma and negative symptoms.

|  | Number of Studies (*k*) | Number of Participants (*n*) | Effect Size (*r*) | 95% CI | *p* | SE | *Z* | *I^2^* | *τ^2^* |
| --- | --- | --- | --- | --- | --- | --- | --- | --- | --- |
| ISMI – All Versions | 65 | 8170 | 0.20 | 0.16 – 0.23 | <0.0001 | 0.02 | 9.9 | 63.5% | 0.01 |
| ISMI – Full Versions Only | 54 | 6876 | 0.19 | 0.15 – 0.23 | <0.0001 | 0.02 | 9.7 | 55.3% | 0.01 |
| PANSS | 46 | 5892 | 0.16 | 0.12 – 0.20 | <0.0001 | 0.02 | 8.1 | 48.3% | 0.01 |

Abbreviations: ISMI: Internalized Stigma of Mental Illness Scale; PANSS: Positive and Negative Syndrome Scale.


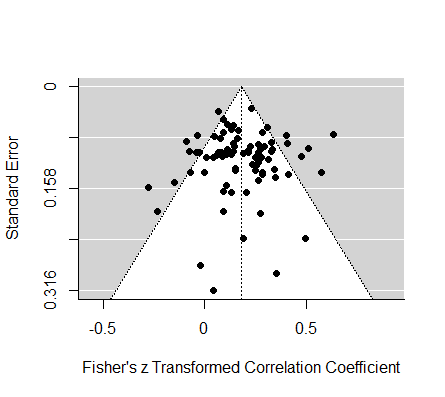


**Figure S18.** Outlier studies excluded: updated funnel plot for primary meta-analysis of internalized stigma.

| Study | N | Country | Diagnosis | Status | Illness Duration – Years *M* (SD) | Age *M* (SD) | % Male | Negative Symptom Severity | Negative Symptom Measure | Perception of Limited Resources Measure |
| --- | --- | --- | --- | --- | --- | --- | --- | --- | --- | --- |
| Bennett et al. (2023) | 105 | USA | SZ/SA | Outpatients | 25.3^a^ | 55.6 (10.6) | 91.4% | 4.8 | CAINS | SARA-Q |
| Bortolon et al. (2014) | 45 | France | SZ |  |  | 34.3 (12.7) | 62% | 2.2 | PANSS | MCQ-CC^b^ |
| Bröcker et al. (2017) | 21 | Germany | SSD+ | Mixed (I) | 13.95 (10.3) | 40.4 (11) | 63.6% | 1.9 | PANSS | MCQ-CC |
| Brüne et al. (2019) | 7 | Germany | SSD |  |  | 23.8 (5.3) | 86.7% | 2.1 | PANSS | MCQ-CC |
| Couture et al. (2011) | 62 | USA | SZ/SA | Outpatients | 24.9^c^ | 46.7 (8.4) | 62.9% | 3.2 | CAINS | SARA-Q |
| Minor et al. (2022) | 20 | USA | SZ | Outpatients |  | 44.3 | 45% | 1.6 | PANSS | MCQ-CC |
| Moritz et al. (2010) | 39 | Germany | SZ |  |  | 34.8 (10.7) | 56.4% | 8.0 | CAPE | MCQ-CC |
| Østefjells et al. (2015)^d^ | 92 | Norway | SSD |  | ≤ 2^e^ | 28.2 (8.3) | 62% | 1.8 | PANSS | MCQ-CC |
| Popolo et al. (2017) | 26 | Italy | SZ | Outpatients |  | 38 (10.1) | 69.2% | 4.9 | BPRS | MCQ-CC |
| Strauss et al. (2015) | 46 | USA | SZ/SA | Outpatients |  | 41.7 (10.7) | 60.9% |  | BNSS | SARA-Q |

**Perception of Limited Resources**

**Table S34.** Summary of studies included in primary meta-analysis for perception of limited resources.

Abbreviations: BNSS: Brief Negative Symptom Scale; BPRS: Brief Psychiatric Rating Scale; CAINS: Clinical Assessment Interview for Negative Symptoms; CAPE: Community Assessment of Psychic Experiences; MCQ-CC: Metacognitions Questionnaire – Cognitive Confidence; Mixed (I): Majority Inpatients; PANSS: Positive and Negative Syndrome Scale; SA: Schizoaffective Disorder; SARA-Q: Success and Resources Appraisals Questionnaire; SSD: Schizophrenia-Spectrum Disorder; SSD+: Schizophrenia-Spectrum Disorder + Other Diagnoses (≤ 15%); SZ: Schizophrenia.

^a^N=103 for illness duration.

^b^65-item version of the MCQ.

^c^Calculated by subtracting mean age of first psychiatric treatment from mean age.

^d^Spearman’s correlation used for effect size.

^e^Maximum two illness episodes or two years of adequate psychosis treatment.

**Table S34.1.** Summary of studies included in subdomain analysis for perception of limited resources.

| Study | N | Diagnosis | Diminished Motivation Measure | Perception of Limited Resources Measure |
| --- | --- | --- | --- | --- |
| Bennett et al. (2023) | 104 | SZ/SA | CAINS-MAP | SARA-Q |
| Couture et al. (2011) | 62 | SZ/SA | CAINS-MAP | SARA-Q |
| Gesraha et al. (2023) | 100 | SZ | MTQ | MCQ-CC |

Abbreviations: CAINS: Clinical Assessment Interview for Negative Symptoms; MAP: Motivation and Pleasure subscale; MCQ-CC: Metacognitions Questionnaire – Cognitive Confidence; MTQ: Motivational Trait Questionnaire; SA: Schizoaffective Disorder; SARA-Q: Success and Resources Appraisals Questionnaire; SZ: Schizophrenia.

Note: *k* < 3 for diminished expression subdomain.

*
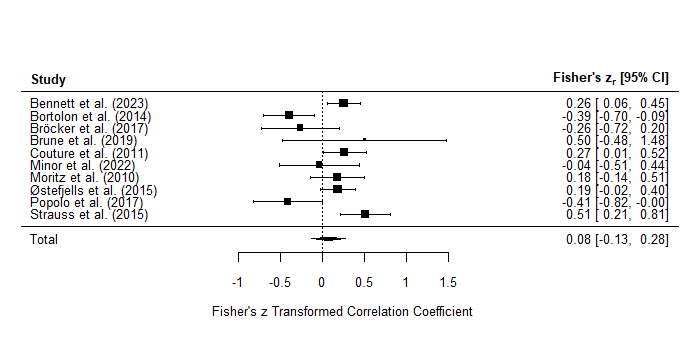
*

**Figure S19.** Forest plot of studies included in primary meta-analysis between perception of limited resources and overall negative symptoms. Effect sizes represented by *z*-transformed Pearson’s *r* correlations.

**Table S35.** Meta-analysis for relationship between perception of limited resources and total negative symptoms, as well as subgroup analysis for diminished motivation subdomain.

|  | Number of Studies (*k*) | Number of Participants (*n*) | Effect Size (*r*) | 95% CI | *p* | SE | *Z* | *I^2^* | *τ^2^* |
| --- | --- | --- | --- | --- | --- | --- | --- | --- | --- |
| Total Negative Symptoms | 10 | 463 | 0.08 | -0.13 – 0.27 | 0.5 | 0.10 | 0.7 | 75.0% | 0.07 |
| Diminished Motivation Subdomain | 3 | 266 | 0.29 | 0.18 – 0.40 | <0.0001 | 0.06 | 4.8 | 0% | 0 |

**Table S36.** Meta-regression analyses of moderators of relationship between overall negative symptoms and perception of limited resources.

|  | Number of Studies (*k*) | Number of Participants (*n*) | ß | SE | *p* |
| --- | --- | --- | --- | --- | --- |
| Age | 10 | 463 | 0.006 | 0.01 | 0.6 |
| Sex (% Male) | 10 | 463 | 0.005 | 0.009 | 0.5 |
| Years of Illness | 3 | 188 | 0.05 | 0.02 | 0.04 |
| Negative Symptom Severity | 9 | 417 | 0.03 | 0.07 | 0.6 |
| Study Quality | 10 | 463 | 0.10 | 0.05 | 0.05 |

**Table S37.** Subset analyses for each measure of perception of limited resources (minimum *k* = 3) included in primary meta-analysis.

|  | Number of Studies (*k*) | Number of Participants (*n*) | Effect Size (*r*) | 95% CI | *p* | SE | *Z* | *I^2^* | *τ^2^* |
| --- | --- | --- | --- | --- | --- | --- | --- | --- | --- |
| MCQ-CC | 7 | 250 | -0.07 | -0.30 – 0.16 | 0.5 | 0.1 | -0.6 | 62.0% | 0.06 |
| SARA-Q | 3 | 213 | 0.31 | 0.17 – 0.42 | <0.0001 | 0.07 | 4.5 | 0.01% | 0 |

Abbreviations: MCQ-CC: Metacognitions Questionnaire – Cognitive Confidence; SARA-Q: Success and Resources Appraisals Questionnaire.

**Table S38.** Subset analyses for each negative symptom measure (minimum *k* = 3) included in primary meta-analysis for internalized stigma.

|  | Number of Studies (*k*) | Number of Participants (*n*) | Effect Size (*r*) | 95% CI | *p* | SE | *Z* | *I^2^* | *τ^2^* |
| --- | --- | --- | --- | --- | --- | --- | --- | --- | --- |
| PANSS | 5 | 185 | -0.07 | -0.34 – 0.22 | 0.7 | 0.1 | -0.4 | 61.2% | 0.06 |

Abbreviations: PANSS: Positive and Negative Syndrome Scale.


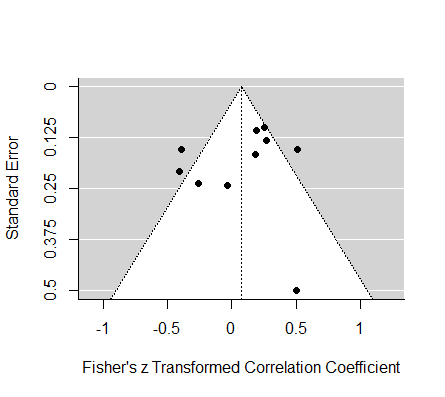


**Figure S20.** Funnel plot for primary meta-analysis of perception of limited resources.


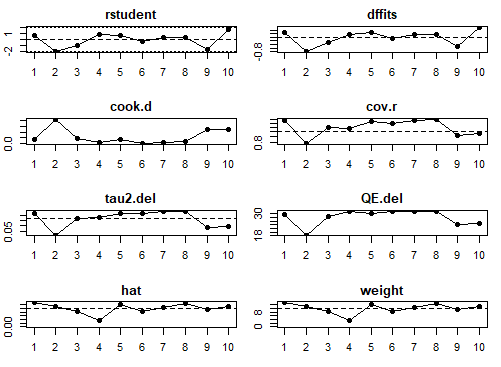


**Figure S21.** Influential outlier analyses for studies included in the primary meta-analysis of perception of limited resources (*k* = 10). Significant studies are illustrated by the presence of red dots.

**References**

Beck, Grant, P. M., Huh, G. A., Perivoliotis, D., & Chang, N. A. (2013). Dysfunctional Attitudes and Expectancies in Deficit Syndrome Schizophrenia. *Schizophrenia Bulletin*, *39*(1), 43–51. https://doi.org/10.1093/schbul/sbr040

Best, M. W., Milanovic, M., Tran, T., Leung, P., Jackowich, R., Gauvin, S., … Bowie, C. R. (2020). Motivation and engagement during cognitive training for schizophrenia spectrum disorders. *Schizophrenia Research: Cognition*, *19*, 100151. https://doi.org/10.1016/j.scog.2019.100151

Campellone, T. R., Sanchez, A. H., & Kring, A. M. (2016). Defeatist Performance Beliefs, Negative Symptoms, and Functional Outcome in Schizophrenia: A Meta-analytic Review. *Schizophrenia Bulletin*, *42*(6), 1343–1352. https://doi.org/10.1093/schbul/sbw026

Chang, Cheung, R., Hui, C. L. M., Lin, J., Chan, S. K. W., Lee, E. H. M., & Chen, E. Y. H. (2015). Rate and risk factors of depressive symptoms in Chinese patients presenting with first-episode non-affective psychosis in Hong Kong. *Schizophrenia Research*, *168*(1), 99–105. https://doi.org/10.1016/j.schres.2015.07.040

Hofer, A., Mizuno, Y., Frajo-Apor, B., Kemmler, G., Suzuki, T., Pardeller, S., … Uchida, H. (2016). Resilience, internalized stigma, self-esteem, and hopelessness among people with schizophrenia: Cultural comparison in Austria and Japan. *Schizophrenia Research*, *171*(1), 86–91. https://doi.org/10.1016/j.schres.2016.01.027

Khalaf, O. O., Fathy, H., Ebrahim, H. A. M., & Samie, M. A. (2023). Self-stigma and coping in youth with schizophrenia and bipolar disorder: A comparative study. *Middle East Current Psychiatry*, *30*(1), 76. https://doi.org/10.1186/s43045-023-00350-0

Lysaker, P. H., Roe, D., & Yanos, P. T. (2007). Toward Understanding the Insight Paradox: Internalized Stigma Moderates the Association Between Insight and Social Functioning, Hope, and Self-esteem Among People with Schizophrenia Spectrum Disorders. *Schizophrenia Bulletin*, *33*(1), 192–199. https://doi.org/10.1093/schbul/sbl016

Magaletta, P. R., & Oliver, J. M. (1999). The hope construct, will, and ways: Their relations with self-efficacy, optimism, and general well-being. *Journal of Clinical Psychology*, *55*(5), 539–551. https://doi.org/10.1002/(SICI)1097-4679(199905)55:5<539::AID-JCLP2>3.0.CO;2-G

Pillny, M., Schlier, B., & Lincoln, T. M. (2020). “I just don’t look forward to anything”. How anticipatory pleasure and negative beliefs contribute to goal-directed activity in patients with negative symptoms of psychosis. *Schizophrenia Research*, *222*, 429–436. https://doi.org/10.1016/j.schres.2020.03.059

Rector, N. A. (2004). Dysfunctional Attitudes and Symptom Expression in Schizophrenia: Differential Associations With Paranoid Delusions and Negative Symptoms. *Journal of Cognitive Psychotherapy*, *18*(2), 163–173. https://doi.org/10.1891/jcop.18.2.163.65959

Santosh, S., & Kundu, P. S. (2023). Cognitive self-efficacy in schizophrenia: Questionnaire construction and its relation with social functioning. *Industrial Psychiatry Journal*, *32*(1), 71. https://doi.org/10.4103/ipj.ipj_263_21

Shaheen, S., & Amin, R. (2016). Effects of internalized stigma on symptoms and quality of life in schizophrenic patients; mediated by dysfunctional attitude. *International Journal of Health*, *4*(2), 145–150. https://doi.org/10.14419/ijh.v4i2.6754
